# EZH2 deficiency suppresses colorectal cancer progression by inhibiting the mismatch repair pathway and consequently reducing extrachromosomal circular DNA formation

**DOI:** 10.1038/s41419-026-08919-3

**Published:** 2026-06-03

**Authors:** Quanpeng Qiu, Yi Ding, Jing Han, Xiaolong Guo, Jiaqi Zhang, Yue Chen, Xinzhu Xue, Xiake Hu, Tianyu Yu, Gaixia Liu, Haowei Zhang, Qixin Li, Xiang Li, Junjun She, Yinnan Chen

**Affiliations:** 1 Department of General Surgery, The First Affiliated Hospital of Xi’an Jiaotong University, Xi’an, Shaanxi China; 2 Department of High Talent, The First Affiliated Hospital of Xi’an Jiaotong University, Xi’an, Shaanxi China; 3 Department of Otolaryngology-Head and Neck Surgery, The First Affiliated Hospital of Gannan Medical University, Ganzhou, Jiangxi China; 4 Center for Gut Microbiome Research, Med-X Institute, The First Affiliated Hospital of Xi’an Jiao tong University, Xi’an, Shannxi China; 5 Shaanxi Belt and Road Joint Laboratory on Gut Microbiome and Cancer, Yulin Hospital, The First Affiliated Hospital of Xi’an Jiao Tong University, Yulin, Shaanxi China; 6 Hubei Key Laboratory of Precision Radiation Oncology, Wuhan, Hubei China

**Keywords:** Cancer genomics, Colorectal cancer

## Abstract

The malignant transition of colorectal cancer (CRC) is caused by the combined action of epigenetic dysregulation and genomic instability. It is well known that EZH2-directed H3K27me3 modification is one of the main contributors to tumorigenesis; however, how it affects the DNA mismatch repair (MMR) system and the genesis of extrachromosomal circular DNA (eccDNA) remains unknown. Here, we demonstrate that EZH2 epigenetically represses critical *MMR* genes, which results in genomic instability and abnormal eccDNA accumulation, ultimately accelerating CRC progression. On the molecular level, the H3K27me3 mark catalyzed by EZH2 is the main cause of the downregulation of *MMR* genes, which affects the stability of the genome and leads to an increase in eccDNA that makes the tumor more aggressive. Our study reveals an unprecedented role of EZH2 in controlling chromosomal instability and eccDNA production through the MMR pathway and offers a conceptual framework for the development of CRC-targeted epigenetic therapies.

## Introduction

Colorectal cancer (CRC) is among the leading types of cancer worldwide [1]. Its development is understood to be a series of complex changes involving both genetic and epigenetic factors [2]. A typical characteristic of CRC is the presence of genomic instability, which primarily manifests in two forms: chromosomal instability (CIN) and microsatellite instability. CIN is characterized by recurrent aneuploidies and structural chromosomal aberrations that drive clonal evolution through enhancing genomic diversity and selecting for aggressive malignant phenotypes [3]. The DNA mismatch repair (MMR) system is essential in preserving genomic integrity by fixing DNA replication errors, and its impairment results in MSI, which is present in ~15% of sporadic CRCs and most Lynch syndrome-related CRCs [4]. Conversely, while MMR deficiency leads to higher mutation rates, a few recent reports indicate that some MMR defects may also affect CIN and the generation of extrachromosomal circular DNA (eccDNA), although the detailed mechanisms are still unclear.

In cancer cells, defects in the MMR system tend to result in an increase in DNA damage and eventually cause errors in DNA replication. This chain of events results in CIN, which often is a source of eccDNA formation [5, 6]. Extrachromosomal DNA plays a major role in tumor evolution, and new studies are showing how large ecDNA (100 kb-1 Mb) and small eccDNA ( <1 kb) together contribute to cancer biology [7, 8]. Despite their modest size, eccDNAs exert unique regulatory functions owing to their preferential enrichment within gene-rich regions and open chromatin domains [9, 10]. Indeed, eccDNAs mostly derive from genomic regions with high transcriptional activity, such as exons, 5’-UTRs, and CpG islands, which hints at their important function in oncogenic gene expression networks [11]. Elevated eccDNA levels are a characteristic of numerous types of cancers, including CRC, where they are particularly concentrated at fragile sites as well as at chromosomal breakpoints associated with CIN. Amplification of genetic elements through eccDNA is one of the mechanisms by which tumors are able to grow rapidly [12]; however, our understanding of the molecular switch governing eccDNA formation is yet limited. The dynamic interaction between extrachromosomal elements and CIN creates a cycle of mutual reinforcement in which chromosomal breaks not only act as a template for eccDNA generation, but the newly formed eccDNAs also aggravate genomic instability via recombination-mediated rearrangements [13, 14]. This linked phenomenon of extrachromosomal inheritance probably forms an important but overlooked aspect of CRC development and clonal evolution.

The biogenesis of eccDNA has also been associated with the intensification of DNA damage, defects in DNA repair pathways, and chromosomal fragmentation resulting in broken DNA fragments, which are usually repaired through nonhomologous or microhomology-mediated end joining [15–17]. Besides MSI, eccDNA also constitutes one of the different types of genomic instabilities that lead to tumor progression and therapeutic resistance in cancer. While these factors independently contribute to the intricacy of tumor biology, the combination of MSI and high eccDNA levels in CRC has not been properly investigated. Understanding the effects of their simultaneous presence may help in the creation of tailored therapeutic approaches for this particular molecular CRC subtype.

Enhancer of zeste homolog 2 (EZH2), which is the enzymatic component of the Polycomb repressive complex 2 (PRC2), controls gene silencing through H3K27me3 and is often abnormally expressed at a high level in CRC [18]. Although EZH2 is known to be an oncogene by enhancing proliferation and metastasis, new findings indicate that its roles depend on the context and may even include tumor-suppressive functions in certain cell environments [19]. Despite EZH2 being very well known for its involvement in DNA repair and genomic stability, it remains unknown how its regulation of the MMR pathway, CIN, and eccDNA formation is carried out.

The present study explores the functional aspects of eccDNA in CRC progression. Mechanistically, EZH2 epigenetically downregulates the MMR pathway, thereby enhancing CIN and resulting in the production of oncogenic eccDNA, which together lead to CRC growth. Additionally, we show that specifically blocking eccDNA generation causes tumor growth inhibition, thus offering a strong theoretical basis as well as a set of highly prioritized therapeutic targets for the design of personalized CRC therapies.

## Materials and methods

### Human tissue samples

All clinical samples were collected from the First Affiliated Hospital of Xi’an Jiaotong University (Xi’an, China), and the diagnosis of CRC was jointly determined by two professors of pathology. Hematoxylin and eosin (H&E) staining was used to quantify the content of tumor cells in tissues. This study was reviewed and approved by the Medical Ethics Committee of the First Affiliated Hospital of Xi’an Jiaotong University (Ethics Lot Number: XJTU1AF2023LSK-2023-218).

### Cell culture

The human embryonic kidney cell line HEK-293T and CRC cell lines HCT116, DLD1, SW480, MC38, and CT26 were kindly provided by Professor Hongping Xia (Department of Pathology, School of Basic Medical Sciences, Nanjing Medical University). The normal colon epithelial cell line NCM460 was purchased from Fuheng Biotechnology Co, LTD (Cat No: FH0541). All cells were identified by STR analysis prior to experimental studies. COLO320 DM and COLO320 HSR cell lines were obtained from ATCC. The cells were cultured at 37 °C in DMEM medium (HEK-293T, HCT116, DLD1, SW480) or RPMI- 1640 medium (NCM460, COLO320 DM, COLO320 HSR, MC38, CT26) supplemented with 10% fetal bovine serum (Gibco; Thermo Fisher Scientific, Waltham, MA, USA) in a humidified incubator containing 5% CO_2_.

### Stable shRNA transfection

The recombinant plasmid was constructed by inserting shRNA into the plasmid vector pLKO.1. Lentiviruses were packaged in HEK-293T cell lines by co-transfection of the recombinant plasmid vectors and helper plasmid vectors pVsvg, pGag/Pol and pRev with lipofectamine 3000 (Invitrogen, Carlsbad, CA, USA). The virus was collected after 48 and 72 hours. After CRC cell lines were infected with the concentrated virus for 48 hours, they were subjected to cell screening by puromycin.

### Stable sgRNA transfection

The recombinant plasmid was constructed by inserting sgRNA into the plasmid vector pLenti-CRISPR V2. To produce lentivirus, HEK-293T cells were seeded overnight. Lentiviral vector pLenti-CRISPR V2 and helper plasmid vectors psPAX2 and pMD2.G were co-transfected into HEK-293T cells using lipofectamine 3000 (Invitrogen, Carlsbad, CA, USA). After puromycin selection, single cells were expanded into subclones.

### Stable transfection of overexpression plasmids

pLV3-CMV-KIF2C vector (Cat. No.P50562) was obtained from MiaoLingBio, China. Recombinant plasmid vector and helper plasmid vectors psPAX2 and pMD2.G were mixed with lipofectamine 3000 (Invitrogen, Carlsbad, CA, USA) and transfected into HEK-293T cell lines.

### RNA extraction and RNA-seq analysis

Total RNA was extracted from cell lines using Trizol reagent (Solarbio, Beijing, China) according to a standard protocol. RNA-seq data were filtered to remove the adaptors and low-quality bases using fastp. Then Salmon and tximeta were used for gene-level quantifications, and the DESeq2 R package for the detection of differentially expressed genes with log2 transformed fold-change >1 and Benjamini–Hochberg adjusted *p* values <0.001.

### Western blot assay

The cells were lysed by RIPA Lysis Buffer (Beyotime, Shanghai, China) with proteinase and phosphatase inhibitors. An equal amount of protein was added to the SDS-PAGE and then transferred to PVDF Blotting Membrane (AmershamTM HybondTM, Amsterdam, The Netherlands). Subsequently, the PVDF Blotting Membrane was blocked with 5% fat-free dry milk at room temperature for 2 hours and incubated with the primary antibodies against β-Actin (1:10000, Proteintech, China), α-Tubulin (1:10000, Proteintech, China), PMS2 (1:1000, Proteintech, China), MSH2 (1:10,00, Proteintech, China), ABCC5 (1:1000, ZenBio, China), KIF2C (1:1000, ZenBio, China), H3 (1:8000, CST, USA), H3K27ac (1:1000, CST, USA), H3K27me3 (1:1000, CST, USA), H3K9ac (1:1000, Active Motif, USA), H3K9me3 (1:1000, CST, USA), H3K4ac (1:1000, Active Motif, USA), H3K4me3 (1:1000, CST, USA), γ-H2A.X (1:1000, Active Motif, USA) at 4 °C overnight. After the incubation, the membrane was washed with 1× TBST three times and incubated with corresponding secondary goat-anti-rabbit IgG (1:10,000, Proteintech, China) or goat anti-mouse IgG antibodies (1:10,000, Proteintech, China) at room temperature for two hours. After rewashing with 1× TBST, the protein bands were detected by Chemiluminescent HRP substrate (EMD Millipore, Boston, MA, USA). Protein expression was semi-quantified using ImageJ version 1.46 software (National Institutes of Health, Bethesda).

### DNA extraction

DNA was extracted using the DNeasy Blood and Tissue kit (Qiagen) according to a standard protocol for high-quality extraction per the manufacturer’s instructions. DNA concentration was determined using a NanoDrop spectrophotometer.

### Circle-sequencing analysis

eccDNA was enriched using the Hispeed® Plasmid Midi Kit (Qiagen) according to a standard protocol. Then FastDigest MssI (Thermo Scientific) and Plasmid-Safe ATP-dependent DNase (Epicenter, Madison, WI, USA) were used to remove mitochondrial circular DNA and the residual linear DNA. The purified samples were used as templates to amplify eccDNA by φ29 polymerase amplification (REPLI-g Midi Kit, QIAGEN, Germany). The amplification reactions were conducted at 30 °C for 16 hours and library preparation was conducted using NEBNext® Ultra II DNA Library Prep Kit for Illumina (New England Biolabs). The sequencing was performed on an Illumina NovaSeq 6000 sequencer with 150 bp paired-end mode following the manufacturer’s instructions.

Raw reads were filtered to remove the adaptors and low-quality bases using fastp, and were mapped to the human genome reference (GRCh38) using BWA-MEM. Then, we use Circle-Map to detect and the circular DNA, which was further annotated by bedtools. To obtain reliable results, we applied a filter of at least 2 split reads, a circle score greater than 50, and a coverage ratio greater than 0.33 in both the start and end of the circular DNA. EPM is the number of eccDNA per million mapped reads and is normalized according to the sequencing depth of each sample. If any eccDNA in a sample contains a gene, we consider the gene to be eccDNA positive in the sample, it is eccDNA negative. In order to evaluate the impact of eccDNA at the gene level, for each protein-coding gene, we calculated whether it is eccDNA positive and the proportion of eccDNA positive in MSI and MSS samples. The significance of differences in gene frequencies between groups was assessed by Fisher’s exact test.

### EccDNA tracking system

The procedures of the eccDNA tracking system (ecTag) were referred to previously reported [20]. In short, 3 × 10^5^ cells were plated into a glass-viewing area of a confocal dish for 24 hours, transfected with ecTag plasmid vectors dCas9, sgRNA-PUFBSa, Clover-PUFa, with Lipofectamine 3000. After 24 hours, the media was changed to fresh media. After 48 hours post-transfection, the dishes were briefly rinsed with 1× PBS three times and fixed with 4% formaldehyde. Finally, DAPI staining was performed, and the images were collected under a microscope.

### Breakpoint-specific PCR for eccDNA validation

The eccDNA validation was performed by outward PCR with denoted primers, which were designed based on the breakpoint site. The PCR products were loaded on a 1% agarose gel for electrophoresis.

### CUT&RUN assay

Cleavage Under Targets and Release Using Nuclease (CUT&RUN) assays were performed using the CUT&RUN Assay Kit (Cell Signaling Technology, USA) according to the manufacturer’s instructions. Briefly, cells were harvested, washed, and bound to concanavalin A-coated magnetic beads. After permeabilization, cells were incubated with primary antibodies against H3K27me3 (1:50, CST, USA), H3K27ac (1:50, CST, USA), EZH2 (1:50, CST, USA), or normal rabbit IgG (1:50, CST, USA) as a negative control overnight at 4 °C. Following antibody binding, protein A/G-Micrococcal Nuclease (pA/G-MNase) fusion protein was added to digest chromatin at the antibody-targeted sites. The reaction was activated by the addition of CaCl₂, and digested fragments were released into the supernatant. DNA was extracted using phenol-chloroform and purified for downstream analysis.

### Micronuclei staining

3 × 10^5^ cells were seeded in six-well plates, and 1/10 volume of DAPI solution was added to the cell culture medium. Then the cells were cultured at a constant temperature of 37 °C for 10 minutes. Finally, the cells were washed with PBS, and the images were collected under a microscope.

### Karyotyping analysis

Cells in the middle phase of mitosis treated with colchicine (TargetMol, USA)were collected, resuspended in a hypotonic solution, then dropped the pieces and dried in an 80 °C oven. Finally, Giemsa staining was performed and observed under a microscope.

### Apoptosis assay

Cells were collected using EDTA-free trypsin and stained with fluorescein isothiocyanate Annexin V and propidium iodide (PI), and then detected by flow cytometry (KeyGen Biotech, China). Data were analyzed with NovoExpress software (Agilent, USA).

### TUNEL assay

Tumor tissues were dewaxed and hydrated, then fixed with 4% paraformaldehyde and Proteinase K. TUNEL reaction solution was prepared for staining according to the manufacturer’s instructions for the TUNEL Apoptosis Kit (UElandy, China). After adding TUNEL reaction solution to each sample area, DAPI staining was performed, and the images were collected under a fluorescence microscope.

### Immunohistochemistry (IHC) staining and scoring system

Formalin-fixed paraffin-embedded tissue sections were first dewaxed with xylene, then dehydrated with 100% alcohol, 85% alcohol, and 75% alcohol, followed by distilled water. The tissue was placed in citrate buffer (pH 6.0) for epitope repair. Tissue sections were rinsed in PBS buffer. After blocking with 3% bovine serum albumin (BSA), the tissue was incubated with the primary antibody against Ki-67 (1:200, Proteintech, China), γ-H2A.X (1:200, Active Motif, USA) overnight, and then incubated with the secondary antibody (1:200; Proteintech; China) at room temperature. Finally, the tissues were stained with Harris hematoxylin. The immunoreactive score (IRS) was used to quantify the IHC staining, ranging from 0 to 12 as a result of the multiplication of positive cell proportion scores (0–4) and staining intensity scores (0–3). The IRS was calculated as a multiple of these two scores. IHC staining and statistical analysis results were independently evaluated by two professors of pathology, who were blinded to the experimental groups.

### Immunofluorescence (IF) staining

Cells were cultured in 24-well plates for 24 hours, and then fixed with 4% formaldehyde and blocked with the mixture of PBS, 1% BSA, 0.1% Triton X-100 followed by incubation with the primary antibody against γ-H2A.X (1:200, Active Motif, USA). The obtained cells were further incubated CoraLite594-conjugated Goat Anti-Rabbit IgG (1:200; Proteintech; China). Finally, DAPI staining was performed, and the images were collected under a fluorescence microscope.

### Subcutaneous homograft tumors

Five-week-old male C57BL/6 mice were obtained from the experimental animal center of Xi’an Jiaotong University (Xi’an, China). All mice were maintained under SPF housing with a maximum of five mice per cage. Vector cells and shPMS2 single-cell suspensions cells were subcutaneously injected into the dorsal flanks of the mice (*n* = 10 per group). When tumors reached a volume of 100–150 mm^3^, mice were treated as follows: (1) 0.1% DMSO in PBS by intraperitoneal injection twice per week ; (2) 2 mg/kg EPZ6438 by oral gavage twice per week; (3) 2 mg/kg HU by oral gavage twice per week; (3) 2 mg/kg EPZ6438 by oral gavage twice per week + 2 mg/kg HU by oral gavage twice per week. Tumors were measured twice a week and calculated as 0.5 × length × width2. After four or five weeks, the mice were euthanized, and the tumors were excised for further experiments.

### AOM/DSS-induced CRC model

Nine-week-old male C57BL/6 mice were first intraperitoneally injected with a single dose of 10 mg/kg AOM (azoxymethane, Aladdin, Shanghai, China), followed by three cycles of 1.5% DSS (cycle 1), 1.8% DSS (cycle 2) and 1.8% DSS (cycle 3) (dextran sulfate sodium, MP Biomedicals, Solon, Ohio, USA) administration to induce colorectal tumors. After three cycles of administration, mice were treated with the drugs. At the end of the experiment, the mice were euthanized and the tumor number in colorectal were recorded. All animal experiments were approved by the Institutional Animal Care and Use Committee of Xi’an Jiaotong University (Ethics Lot Number: 2023-22).

### Statistical analysis and reproducibility

All data were presented as the means ± standard deviation, and statistical analyses were performed by the GraphPad Prism (version 8, San Diego, CA, USA) with Student’s *T*test. For all analyses, *p* < 0.05 was considered statistically significant. Representative images of micrographs were shown to represent reproducible data from various experiments using micrographs.

## Results

### Aberrant eccDNA biogenesis defines the MSI CRC landscape

**Fig. 1:**
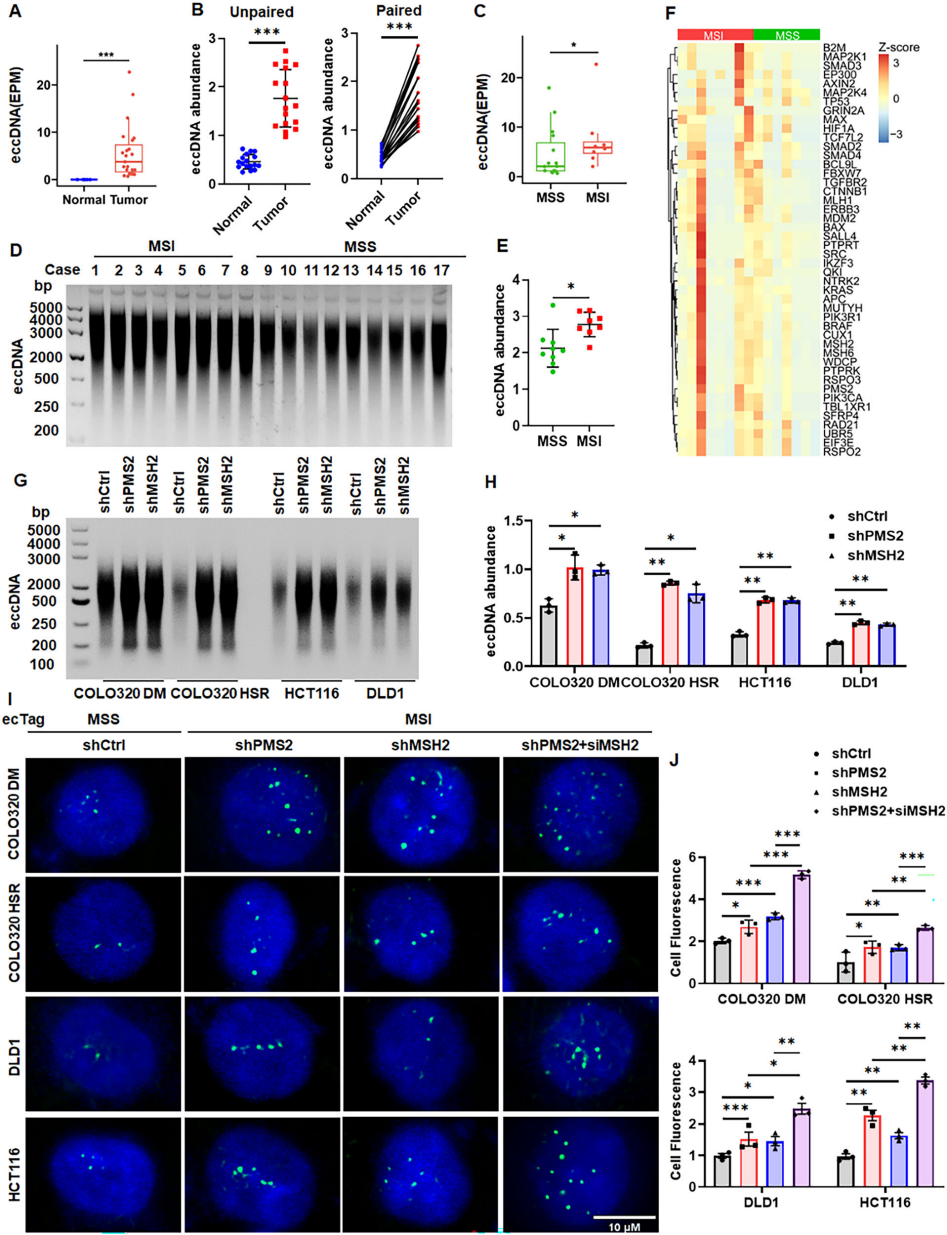
EccDNA is associated with the MSI/MSS subtyping of CRC. **A** EccDNA abundance after removal of linear DNA in CRC tumor and normal tissues analyzed by Circle-seq. **B** EccDNA abundance after removal of linear DNA in CRC tumor and normal tissues was detected by agarose gel electrophoresis. **C** EccDNA abundance after removal of linear DNA in MSI and MSS subtype CRC samples analyzed by Circle-seq. **D** EccDNA abundance after removal of linear DNA in MSI and MSS subtype CRC samples detected by agarose gel electrophoresis. **E** Quantification of eccDNA agarose gel electrophoresis in metaphase of MSI and MSS subtype CRC samples. **F** Heatmap of differential expression of oncogenes in MSI and MSS subtype CRC samples analyzed by Circle-seq. **G** EccDNA abundance after removal of linear DNA in MSI-model cells and wild-type cells detected by agarose gel electrophoresis. **H** Quantification of eccDNA agarose gel electrophoresis in metaphase of MSI-model cells and wild-type cells. **I** Representative ecTag images in metaphase of MSI-model cells and wild-type cells. The oligos target the junction of ecMAPK1. **J** Quantification of ecTag in metaphase of MSI-model cells and wild-type cells. Data were statistically analyzed using a Student *t* test. *p < 0.05, **p < 0.01, ***p < 0.001.

To characterize the eccDNA landscape in CRC, we conducted Circle-seq analysis on 23 tumor tissues as well as five matched adjacent normal tissues from CRC patients (Supplementary Table 1). We noticed a significant increase in eccDNAs in tumor samples compared to their adjacent non-tumor counterparts (Fig. 1A, B; Supplementary Fig. 1A). Based on the eccDNA repertoires identified by Circle-seq, we selected eccMAPK1, a highly abundant eccDNA in our dataset, for downstream validation (Supplementary Table 2). Breakpoint-specific oligos were designed for ecTag assays, which confirmed the presence of eccDNA across a panel of CRC cell lines (COLO320DM, COLO320HSR, HCT116, DLD1, and SW480), while it remained undetectable in normal colon epithelial controls (Supplementary Fig. 1B, C).

To determine subtype-specific patterns, we investigated the eccDNA profiles of microsatellite instability-high (MSI-H) versus microsatellite stable (MSS) tumors using Circle-seq. Our data revealed that MSI-H tumors (*n* = 8) harbor a three-fold greater abundance of eccDNAs than MSS tumors (*n* = 15) (Fig. 1C). This finding was confirmed by the physical separation of circular DNA via agarose gel electrophoresis (Fig. 1D, E). Moreover, our study revealed that several oncogenes, such as B2M, MAP2K1, and SMAD3, were highly represented in the eccDNA pool of CRC tumors (Fig. 1F). Genomic alignment of eccDNA sequences showed broad autosomal representation with significant depletion on the Y chromosome (ChrY) (Supplementary Fig. 1D). This uneven distribution suggests that eccDNA biogenesis may be biased toward gene-rich autosomes rather than the relatively gene-poor and heterochromatic ChrY.

To mechanistically investigate the role of MMR deficiency in eccDNA biogenesis, we established a cellular model of MSI CRC by depleting the MMR genes PMS2 and MSH2 (Supplementary Fig. 2A–C). We observed a significant expansion of the eccDNA repertoire in the MSI-model group (Supplementary Fig. 2D). Genomic alignment confirmed that these eccDNAs originated from all chromosomes except ChrY (Supplementary Fig. 2E), consistent with our clinical observations. Circle-seq analysis of this model revealed the extrachromosomal amplification of key oncogenic drivers, including SMAD4, AXIN2, and TP53 (Supplementary Fig. 1F). The physical abundance of eccDNA was then evaluated in MSI and MSS contexts via eccDNA agarose gel electrophoresis. Remarkably, the loss of MSH2 and PMS2 led to a robust upregulation of eccDNA levels (Fig. 1G, H). Validation via ecTag assays further confirmed that eccDNA frequency was significantly elevated in MSI-model cells compared to wild-type controls (Fig. 1I, J). Collectively, these independent experimental lines demonstrate that MMR deficiency promotes eccDNA accumulation, suggesting a fundamental mechanistic link between DNA repair defects and extrachromosomal DNA amplification in CRC.

### CIN drives eccDNA biogenesis in MSI CRC

**Fig. 2:**
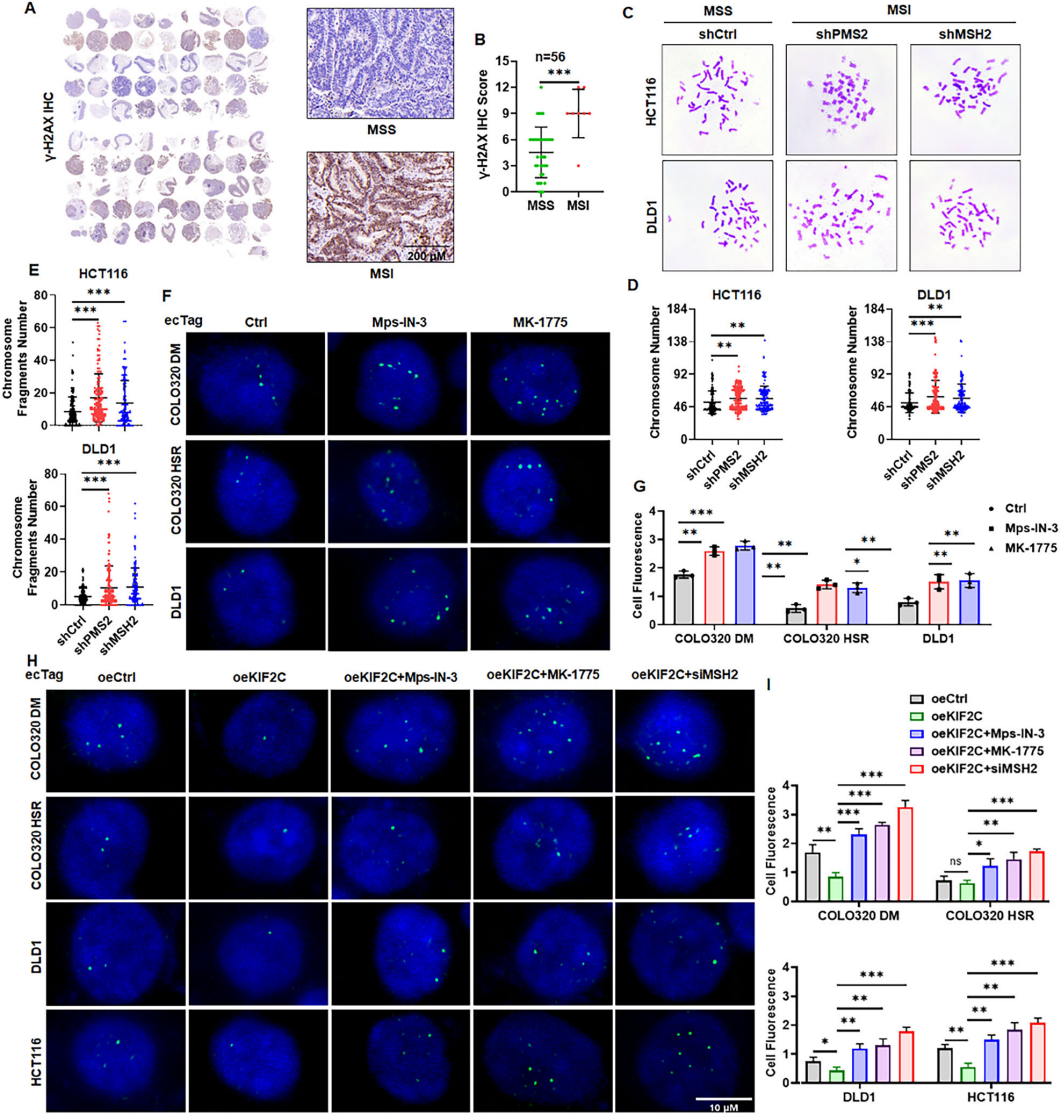
Microsatellite instability in colorectal cancer is associated with increased chromosomal instability. **A** Tissue chips analysis (left) and representative images (right) of γ-H2AX IHC staining in CRC tumor and normal tissues. **B** γ-H2AX IHC score in MSI and MSS CRC samples. **C** Representative images of karyotyping in MSI-model cells and wild-type cells. **D** Numerical chromosome abnormalities in MSI-model cells and wild-type cells. **E** Chromosomal fragmentation in MSI-model cells and wild-type cells. **F** Representative ecTag images of metaphase of CRC cells after treatment with Mps-IN-3 and MK1775. The oligos target the junction of ecMAPK1. **G** Quantification of ecTag in metaphase of CRC cells after treatment with Mps-IN-3 and MK1775. **H** Representative ecTag images in metaphase of oeCtrl cells and oeKIF2C cells after treatment with Mps-IN-3, MK-1775 and both overexpression of KIF2C and knockdown of MSH2 gene. The oligos target the junction of ecMAPK1. **I** EccDNA abundance after removal of linear DNA in metaphase of oeCtrl cells and oeKIF2C cells after treatment with Mps-IN-3, MK-1775 and both overexpression of KIF2C and knockdown of MSH2 gene detected by agarose gel electrophoresis. Data were statistically analyzed using a Student *t* test. *p < 0.05, **p < 0.01, ***p < 0.001.

To elucidate the interplay between CIN and eccDNA formation across molecular CRC subtypes, we performed a tissue microarray analysis of 56 CRC specimens (MSI-H, *n* = 8; MSS, *n* = 48) using immunohistochemistry (Fig. 2A). Compared to the MSS tissues, the DNA damage marker γ-H2AX was significantly enriched in the MSI-H subtype (*P* < 0.001) (Fig. 2B). This finding was corroborated in our MSI cellular model, established by MSH2 and PMS2 depletion, as evidenced by immunofluorescence staining (Supplementary Fig. 3A, B). Furthermore, we observed a marked increase in micronuclei formation in MSI-H cells relative to controls (Supplementary Fig. 3C, D). The karyotypic analysis (Fig. 2C) confirmed an increase in numerical chromosomal abnormalities (Fig. 2D) and extensive chromosomal fragmentation (Fig. 2E) in the MSI-H CRC model, underscoring the high level of CIN associated with MMR deficiency. To investigate the causal relationship between CIN and eccDNA biogenesis, we chemically induced a high-CIN state (CIN-H) in CRC cell lines by treatment with Mps-IN-3 and MK1775. The ecTag assays revealed that ecMAPK1 was significantly enriched in CIN-H cells (Fig. 2F, G). Furthermore, eccDNA agarose gel electrophoresis confirmed a substantial increase in overall eccDNA abundance following the induction of CIN-H (Supplementary Fig. 3E, F). These findings provide direct experimental evidence that induced CIN drives eccDNA formation, mechanistically linking CIN to the eccDNA biogenesis characteristic of MSI CRC tumors.

KIF2C overexpression has been reported to inhibit chromosome missegregation and thereby improve chromosomal stability [21, 22]. Therefore, to study whether chromosomal integrity has any effect on eccDNA dynamics, we used chromosomal stabilization as a functional tool to assess how it affects eccDNA accumulation. Since isogenic pairs of CRC cell lines were not available, we first constructed a chromosomal instability-low (CIN-L) cell model overexpressing KIF2C [23] (Supplementary Fig. 4A). Karyotyping showed that the CIN-L model had a lower percentage of cells with fragmented chromosomes (Supplementary Fig. 4B, C). Moreover, a significant decrease in micronuclei formation was found in CIN-L cells compared to wild-type controls (Supplementary Fig. 4D, E). Consequently, these results confirm KIF2C as a potent determinant of the structural chromosomal stability, which grants an experimental model to examine how chromosomal integrity relates to eccDNA biogenesis. The ecTag analysis was performed to assess ecMAPK1 abundance following treatment with the replication stressor hydroxyurea (HU) in CIN-L and control cells. This treatment resulted in a significant downregulation of ecMAPK1 in CIN-L cells, which recapitulated the effect of HU treatment alone (Supplementary Fig. 5A, B). Also, eccDNA agarose gel electrophoresis showed that global eccDNA levels were reduced along with HU exposure (Supplementary Fig. 5C, D). After further treatment with Mps-IN-3 and MK1775, ecMAPK1 levels in CIN-L cells were partially restored (Fig. 2H, I). Remarkably, this phenotypic recovery was achieved through either KIF2C overexpression or MSH2 depletion (Fig. 2H, I). Collectively, these findings demonstrate that chromosomal stability is a primary determinant of eccDNA biogenesis.

### eccDNA clearance abrogates CRC cell proliferation and survival

**Fig. 3:**
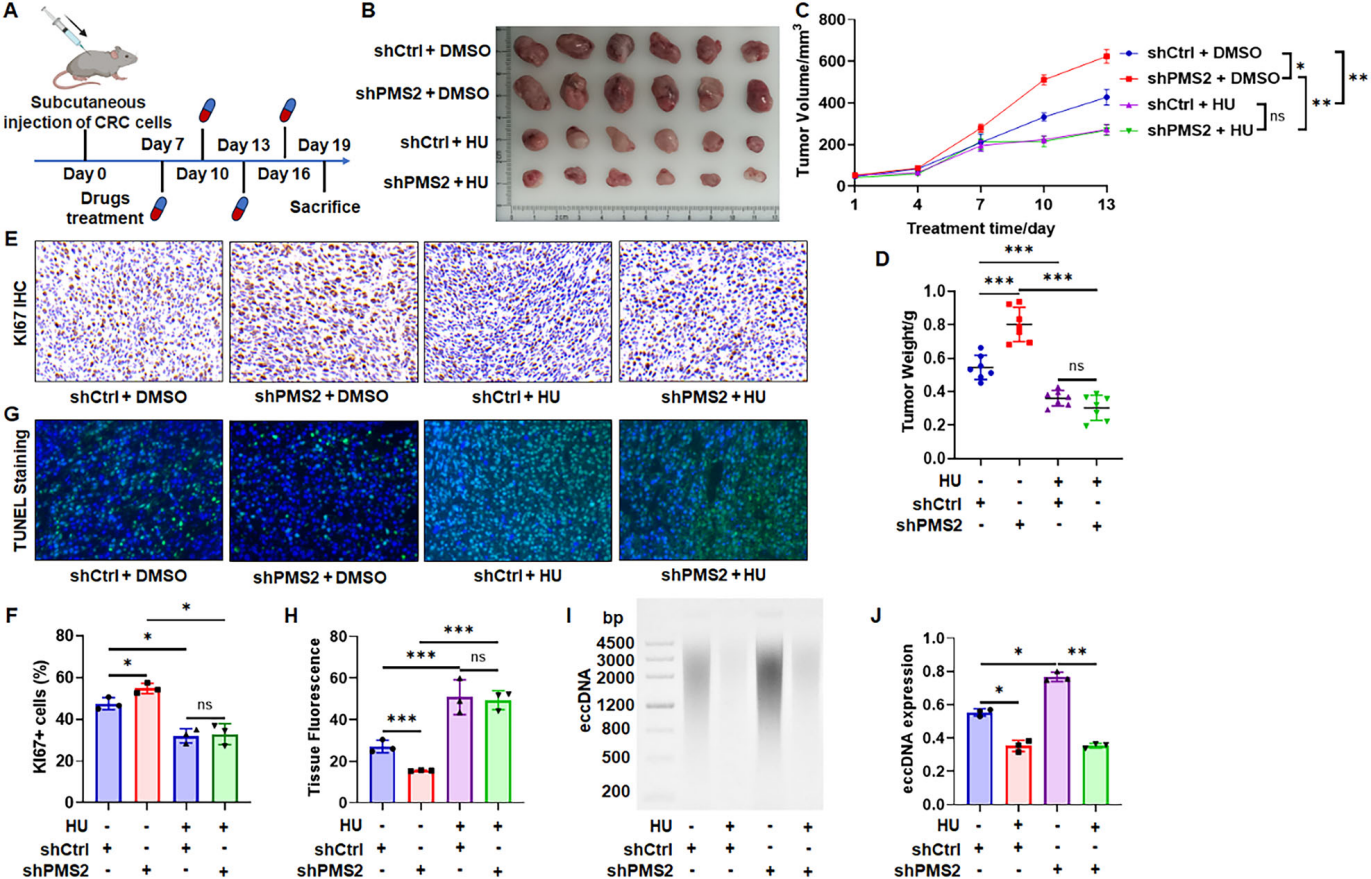
Hydroxyurea treatment reduced the tumor burden of CRC. **A** A schematic diagram of the construction and treatment of subcutaneous homograft tumors in C57BL/6 mice. **B** Representative images of subcutaneous xenograft tumors in shCtrl group and shPMS2 group after treatment with HU (2 mg/kg, oral gavage twice per week). **C** Tumor volume changes in shCtrl group and shPMS2 group after treatment with HU. **D** Tumor weight changes in shCtrl group and shPMS2 group treatment with HU. **E** Representative images of Ki-67 IHC staining in tumor sections. **F** Quantification of Ki-67 IHC staining in tumor sections. **G** Representative images of TUNEL fluorescence staining in tumor sections. **H** Quantification of TUNEL fluorescence staining in tumor sections. **I** EccDNA abundance after removal of linear DNA detected by agarose gel electrophoresis in shCtrl group and shPMS2 group after treatment with HU. **J** Quantification of eccDNA agarose gel electrophoresis in shCtrl group and shPMS2 group after treatment with HU. Data were statistically analyzed using a Student *t* test. *p < 0.05, **p < 0.01, ***p < 0.001, ns: none significance.

To explore the role of eccDNA in cancer progression, we developed an in vivo tumor model by inoculating C57BL/6 mice with PMS2-deficient MC38 cells (Fig. 3A). As such, PMS2 knockdown resulted in faster tumor cell proliferation. On the contrary, treating cells to deplete eccDNA using HU effectively reduced tumor formation (Fig. 3B), as shown by clear decreases in both tumor volume (Fig. 3C) and weight (Fig. 3D). Further mechanistic studies indicated that PMS2 deficiency led to aggressive oncogenic cell growth (Fig. 3E, F) and a reduction in programmed cell death (Fig. 3G, H), which were completely counteracted by HU treatment. Furthermore, the administration of HU drastically decreased eccDNA levels in the tumor microenvironment (Fig. 3I, J). Overall, these results reveal the essential role of eccDNA in promoting cancer progression and suggest that targeting eccDNA has the potential to be an effective anti-cancer therapy.

### EZH2-mediated H3K27me3 deposition induces epigenetic silencing of *MMR* genes

**Fig. 4:**
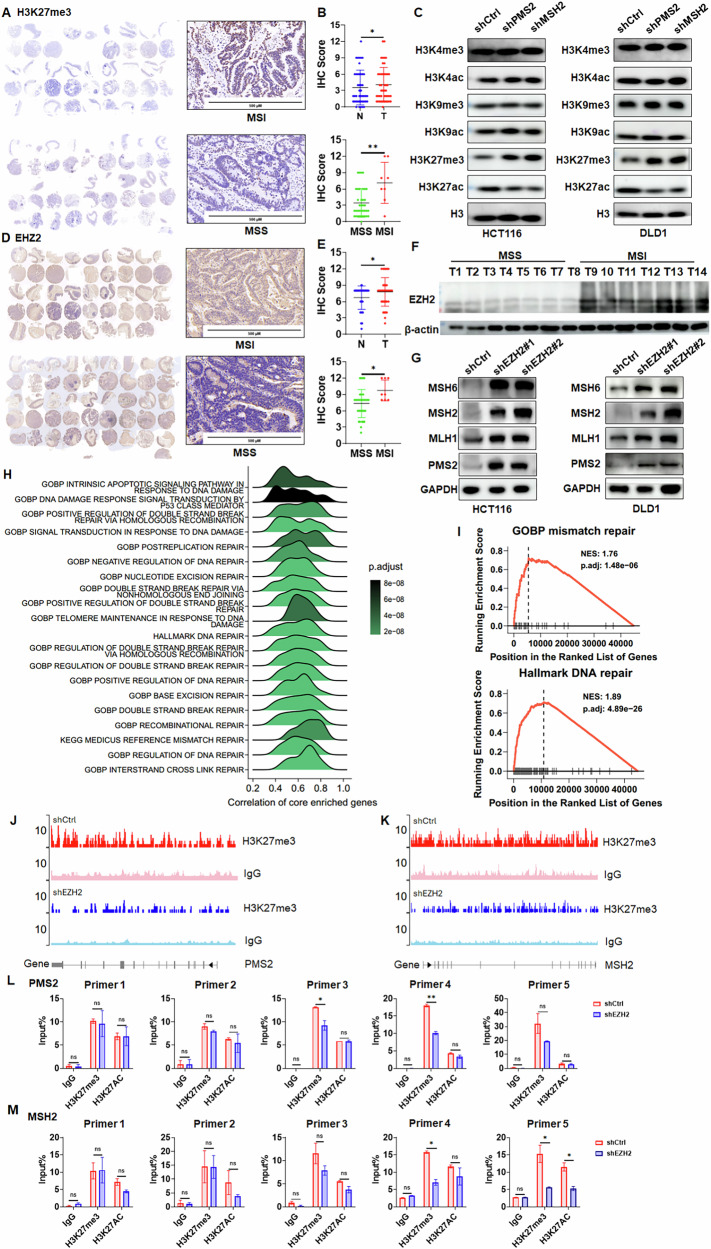
EZH2 regulates the function of MMR pathway. **A** Tissue chips analysis (left) and representative images (right) of H3K27me3 IHC staining in CRC tumor and normal tissues. **B** H3K27me3 IHC score in MSI and MSS CRC samples. **C** The protein expression levels of differential histone post-translational modifications in MSI-model cells and wild-type cells. **D** Tissue chips analysis (left) and representative images (right) of EZH2 IHC staining in CRC tumor and normal tissues. **E** EZH2 IHC score in MSI and MSS CRC samples. **F** The protein expression levels of EZH2 in MSI and MSS CRC samples. **G** The protein expression levels of EZH2 after CRISPR-mediated EZH2 knockdown. **H** Transcriptomic analysis reveals enrichment of DNA damage repair-related pathways. **I** Enrichment analysis of the mismatch repair pathway and the DNA repair pathway in transcriptomic data. **J** CUT&RUN-seq analysis of H3K27me3 enrichment at the PMS2 gene locus in shCtrl and shEZH2 cells. **K** CUT&RUN-seq analysis of H3K27me3 enrichment at the MSH2 gene locus in shCtrl and shEZH2 cells. **L** Validation of binding of H3K27me3 and H3K27ac to the promoter region of PMS2 gene by CUT&RUN qPCR. **M** Validation of binding of H3K27me3 and H3K27ac to the promoter region of MSH2 gene by CUT&RUN qPCR. Data were statistically analyzed using a Student *t* test. *p < 0.05, **p < 0.01, ***p < 0.001, ns: none significance.

Changes in epigenetic hallmarks (i.e., DNA methylation and histone modifications) frequently compromise key MMR genes in the MSI-H subtype [2, 24]. To further understand the epigenetic features of the MMR system, we extensively analyzed the main histone modifications (H3K27me3, H3K27ac, H3K9me3, and H3K9ac) in MSI-H versus MSS CRC tissues. Immunohistochemical staining revealed a substantially higher level of H3K27me3 in MSI-H CRC in comparison to MSS CRC cases (Fig. 4A, B), whereas no statistically significant differences were observed for H3K27ac, H3K9me3, or H3K9ac (Supplementary Fig. 6A–F). Mechanistically, the targeted depletion of PMS2 and MSH2 in CRC cell lines triggered a significant elevation in H3K27me3 levels, accompanied by a reciprocal decrease in H3K27ac (Fig. 4C). Remarkably, other marks, including H3K9me3, H3K9ac, H3K4me3, and H3K4ac, remained unaffected following MMR knockdown (Fig. 4C). These results suggest that H3K27me3 serves as a specific epigenetic regulator involved in the functional modulation of the MMR system in MSI-H CRC.

In order to understand the molecular pathways that modulate H3K27me3 enrichment in MSI-H CRC, we focused on EZH2, the enzymatic component of the PRC2, which is responsible for the trimethylation of H3K27 [18, 19]. Immunohistochemical analysis of our CRC tissue microarray cohort revealed a marked upregulation of EZH2 protein expression in MSI-H relative to MSS tumors (Fig. 4D). Quantitative assessment confirmed significantly higher EZH2 levels in MSI-H CRC tissues (Fig. 4E), a finding corroborated by Western blot analysis (Fig. 4F). We conducted a Spearman correlation on tissue microarray-derived IHC scores to precisely understand the regulatory relationship between EZH2 and histone modifications. As a result, we found that the expression of EZH2 is significantly positively correlated with the levels of both H3K27me3 and H3K9me3, whereas no significant correlations were observed with H3K27ac or H3K9ac (Supplementary Fig. 6G).

To functionally validate the EZH2-MMR axis, we generated stable EZH2-knockdown CRC cell models using CRISPR-Cas9 technology. Western blot analysis demonstrated that EZH2 depletion significantly restored the protein abundance of key MMR components, including PMS2, MSH2, MSH6, and MLH1 (Fig. 4G). Transcriptomic profiling via RNA sequencing (RNA-seq) further showed a significant enrichment of DNA damage repair-related pathways (Fig. 4H), with the MMR pathway showing marked activation following EZH2 silencing (Fig. 4I). Altogether, these findings suggest that EZH2 is a direct epigenetic repressor of the MMR system in CRC.

To elucidate how EZH2 regulates the MMR system at the molecular level, we performed CUT&RUN assays to identify the distribution of H3K27me3 and H3K27ac marks at the promoter regions of PMS2 and MSH2 genes. CUT&RUN sequencing revealed a significant depletion of the repressive H3K27me3 mark at PMS2 and MSH2 promoters following EZH2 knockdown (Fig. 4J, K). This was corroborated by CUT&RUN-qPCR using five independent primer pairs targeting the identified promoter segments, which consistently showed a significant reduction in H3K27me3 occupancy (Fig. 4L, M). These data collectively demonstrate that EZH2 modulates the MMR system by promoting H3K27me3 deposition at the promoter regions of PMS2 and MSH2, thereby modulating their transcriptional activity. To establish whether EZH2 directly orchestrates this repressive landscape, we performed ChIP-qPCR, which demonstrated robust recruitment of EZH2 to the same promoter regions compared to the IgG control (Supplementary Fig. 7A, B). Collectively, the convergence of direct EZH2 binding, localized H3K27me3 deposition, and subsequent transcriptional repression serves as strong evidence that EZH2 directly targets and epigenetically silences *MMR* genes in CRC.

### EZH2 inhibition abrogates eccDNA-driven tumor progression

**Fig. 5:**
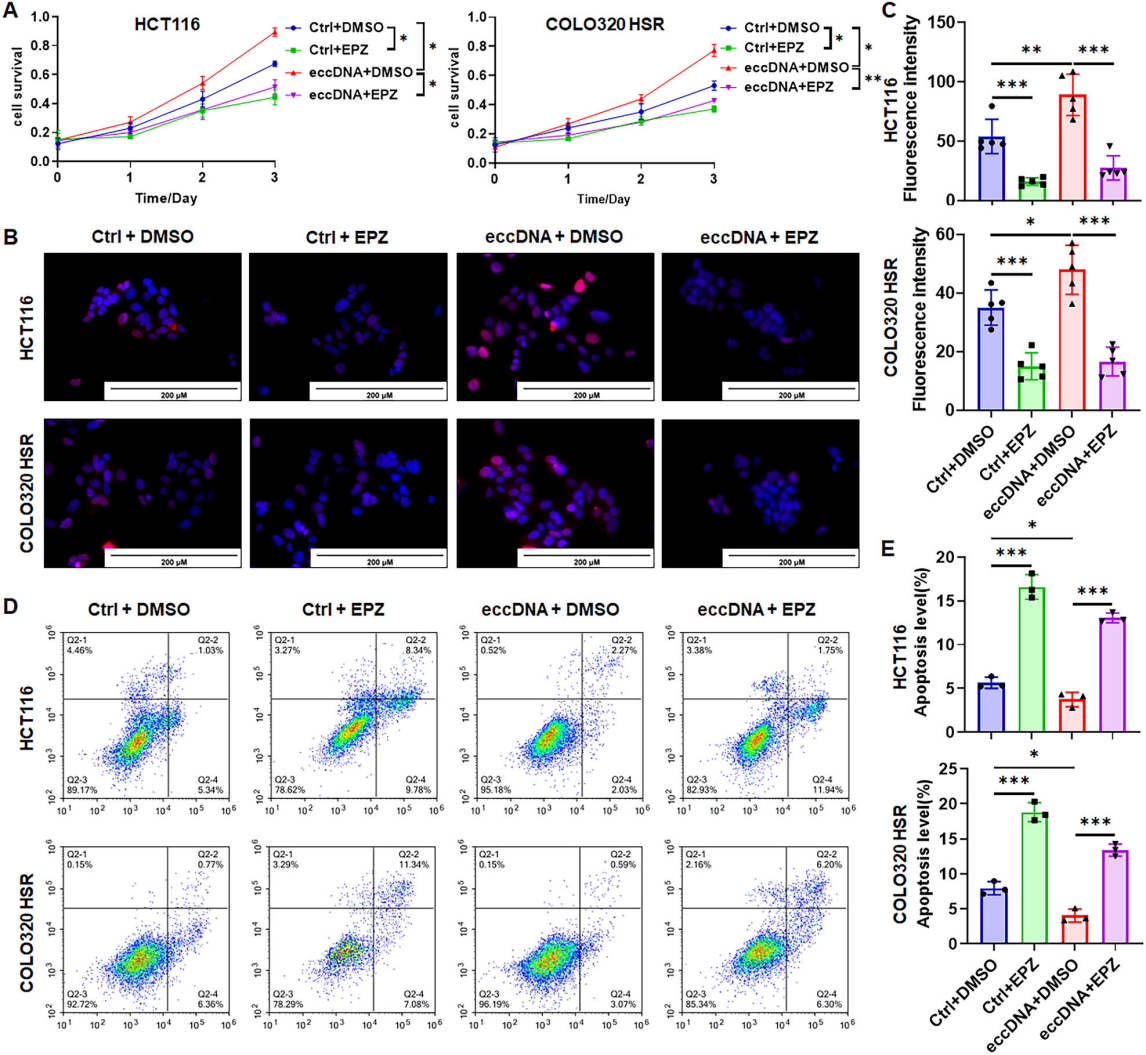
EccDNA accumulation promotes tumor cell proliferation. **A** Proliferation rates of control and eccDNA-transfected CRC cells treated with or without EPZ6438. **B** Representative images of Ki-67 IF staining in control and eccDNA-transfected CRC cells treated with or without EPZ6438. **C** Quantification of Ki-67 IF staining in control and eccDNA-transfected CRC cells treated with or without EPZ6438. **D** Apoptosis of control and eccDNA-transfected CRC cells treated with or without EPZ6438. **E** Quantification of apoptosis in control and eccDNA-transfected CRC cells treated with or without EPZ6438. Data were statistically analyzed using a Student *t* test. *p < 0.05, **p < 0.01, ***p < 0.001, ns: none significance.

To investigate whether oncogenic eccDNAs contribute to CRC development, we extracted and purified eccDNAs from COLO320 DM cells (high eccDNA burden) and then transfected them into COLO320 HSR and HCT116 cells (low eccDNA burden), using empty vectors as negative controls. Proliferation assays over 72 hours showed that the delivery of eccDNAs remarkably promoted cell division, a phenotype that was largely inhibited with the EZH2 inhibitor EPZ6438 (Fig. 5A). This was further corroborated by Ki-67 immunofluorescence staining, which revealed a significantly greater number of Ki-67-positive nuclei in the eccDNA-transfected cells in comparison to the controls (Fig. 5B, C). Moreover, apoptosis analysis revealed a significant decrease in late apoptotic populations within eccDNA-transfected groups, which demonstrates a strong anti-apoptotic effect that was in part reversed by EPZ6438 (Fig. 5D, E). These findings collectively indicate that eccDNA drives tumor progression via dual pro-proliferative and anti-apoptotic effects, which are therapeutically targetable through EZH2 inhibition.

### EZH2 inhibition restores chromosomal integrity and restricts eccDNA biogenesis

**Fig. 6:**
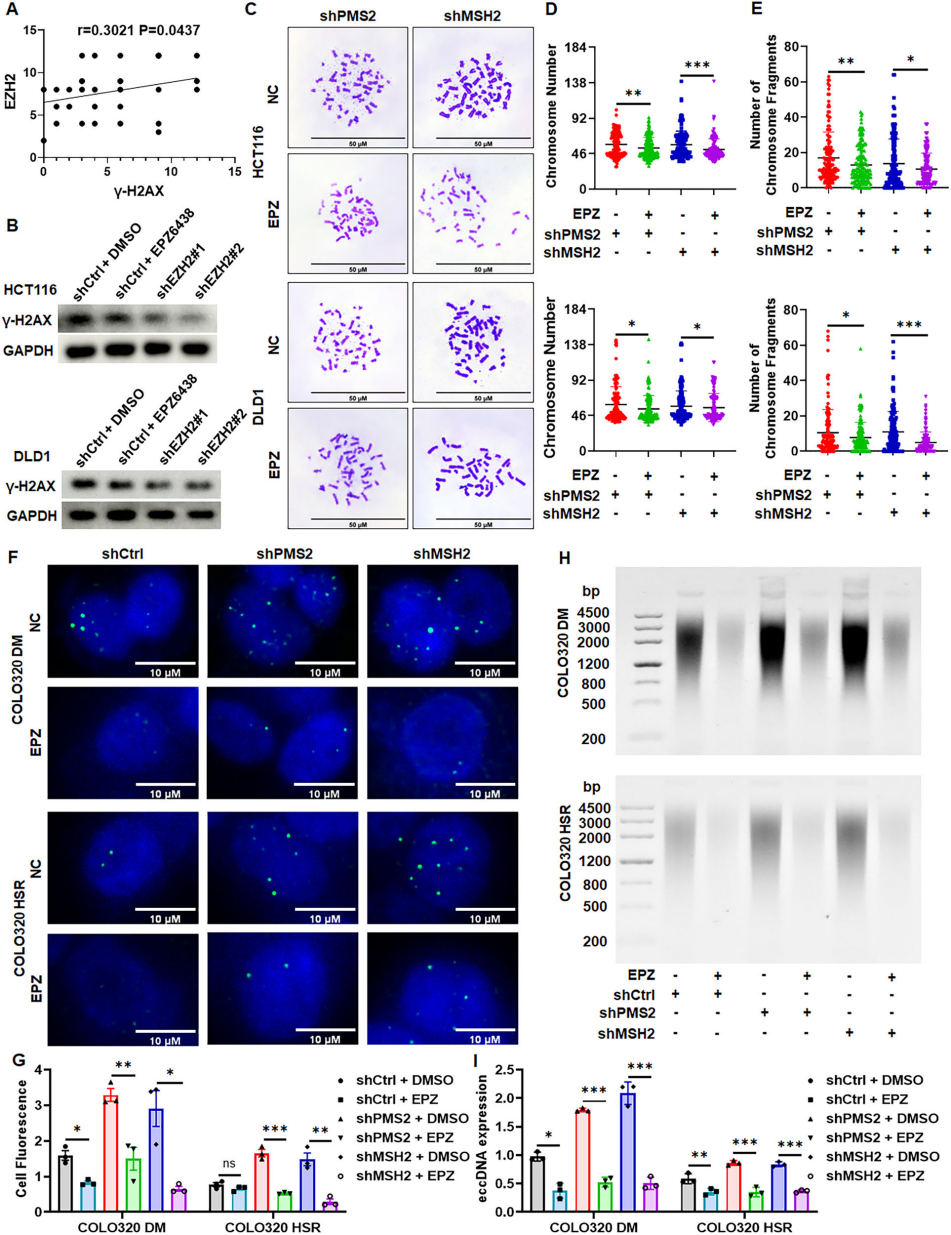
EZH2 inhibition enhances chromosomal stability and reduces eccDNA abundance. **A** Correlation analysis of EZH2 and γ-H2AX expression using tissue microarray. **B** The protein expression levels of γ-H2AX in control and shEZH2 CRC cells treated with or without EPZ6438. **C** Representative images of karyotyping in shPMS2 and shMSH2 CRC cells treated with or without EPZ6438. **D** Numerical chromosome abnormalities in shPMS2 and shMSH2 CRC cells treated with or without EPZ6438. **E** Chromosomal fragmentation in shPMS2 and shMSH2 CRC cells treated with or without EPZ6438. **F** Representative ecTag images in metaphase of MSI-model cells and wild-type cells after treatment with EPZ6438. The oligos targeting the junction of ecMAPK1. **G** Quantification of ecTag in metaphase of MSI-model cells and wild-type cells after treatment with EPZ6438. **H** EccDNA abundance after removal of linear DNA in MSI-model cells and wild-type cells after treatment with EPZ6438, detected by agarose gel electrophoresis. **I** Quantification of eccDNA agarose gel electrophoresis in metaphase in MSI-model cells and wild-type cells after treatment with EPZ6438. Data were statistically analyzed using a Student *t* test. *p < 0.05, **p < 0.01, ***p < 0.001.

As a first step in understanding the regulatory role of EZH2 in eccDNA formation, the clinical relationship between EZH2 and the DNA damage marker γ-H2AX via CRC tissue microarrays. Spearman correlation analysis showed a statistically significant positive correlation between the expression levels of EZH2 and γ-H2AX (Fig. 6A). Western blot analysis demonstrated that, besides EZH2 silencing, pharmacological inhibition of EZH2 with EPZ6438 also decreased γ-H2AX protein expression (Fig. 6B). To characterize CIN, we carried out chromosomal analyses for both numerical and structural abnormalities (Fig. 6C). Our findings showed that EPZ6438-treated cells had significantly decreased frequencies of both numerical (Fig. 6D) and structural (Fig. 6E) chromosomal variations compared to controls. Moreover, micronucleus formation analyses confirmed a decrease in micronucleus formation after EPZ6438 treatment (Supplementary Fig. 8A–D), thus pointing to increased genomic stability. Remarkably, ecTag quantification (Fig. 6F, G) and agarose gel electrophoresis analysis (Fig. 6H, I) demonstrated a significant reduction in eccDNA content in EPZ6438-treated groups. These findings collectively demonstrate that EZH2 regulates eccDNA accumulation by modulating chromosomal stability, and its inhibitor can effectively reduce eccDNA load.

### Synergistic targeting of HU and EZH2 eliminates the eccDNA reservoir

**Fig. 7:**
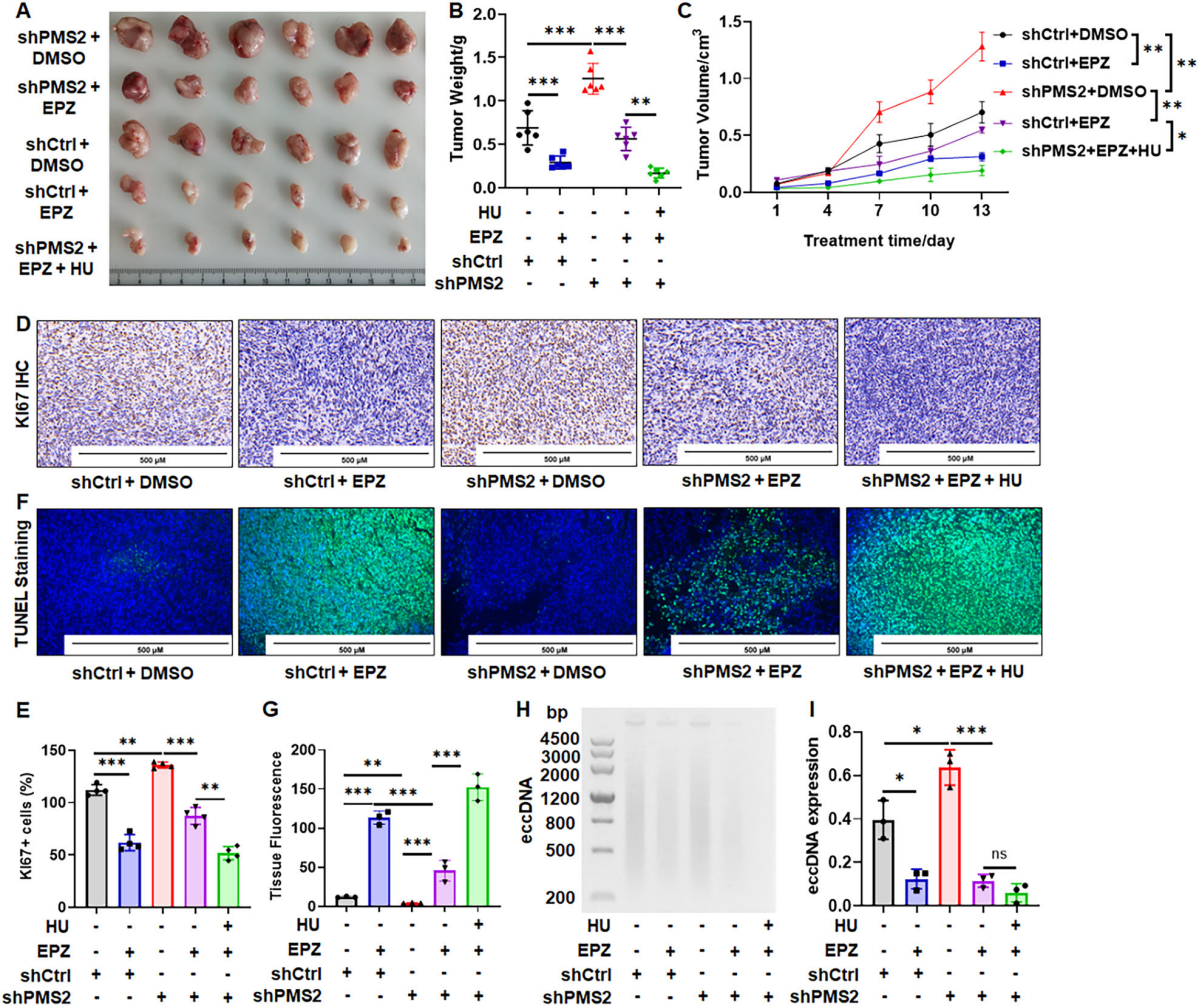
EZH2 inhibitor and hydroxyurea combination reduced the tumor burden of CRC. **A** Representative images of subcutaneous xenograft tumors in shCtrl group and shPMS2 group after treatment with EPZ6438 (2 mg/kg, oral gavage twice per week) and HU (2 mg/kg, oral gavage twice per week). **B** Tumor volume changes in shCtrl group and shPMS2 group after treatment with EPZ6438 and HU. **C** Tumor weight changes in shCtrl group and shPMS2 group treatment with EPZ6438 and HU. **D** Representative images of Ki-67 IHC staining in tumor sections. **E** Quantification of Ki-67 IHC staining in tumor sections. **F** Representative images of TUNEL fluorescence staining in tumor sections. **G** Quantification of TUNEL fluorescence staining in tumor sections. **H** EccDNA abundance after removal of linear DNA detected by agarose gel electrophoresis in shCtrl group and shPMS2 group after treatment with EPZ6438 and HU. **I** Quantification of eccDNA agarose gel electrophoresis in shCtrl group and shPMS2 group after treatment with EPZ6438 and HU. Data were statistically analyzed using a Student *t* test. *p < 0.05, **p < 0.01, ***p < 0.001, ns: none significance.

Since high eccDNA abundance promotes CRC progression, we hypothesized that interfering with eccDNA generation can effectively inhibit tumor growth. To test this, we developed an in vivo model by inoculating C57BL/6 mice with *PMS2*-deficient MC38 cells. Subsequently, animals were treated with EPZ6438 alone or in combination with HU for 14 days. EPZ6438 alone was able to significantly reduce tumor cell proliferation, whereas the combination of EPZ6438 and HU exhibited an enhanced anti-tumor effect, as evidenced by a marked reduction in both tumor volume and weight (Fig. 7A–C). Immunohistochemical analysis showed that the administration of EPZ6438 resulted in a significant decrease in Ki-67 expression levels, with the combination treatment yielding an even stronger blockade of mitotic activity (Fig. 7D, E). Additionally, TUNEL staining revealed an increase in tumor cell apoptosis upon EPZ6438 administration, an effect further amplified by HU co-administration (Fig. 7F, G). Moreover, double targeting with EPZ6438 and HU dramatically lowered the level of eccDNA in tumor tissues (Fig. 7H, I). Collectively, these data indicate that the combination of drugs inhibiting EZH2 and targeting eccDNA represents a promising synergistic approach for CRC treatment.

### Synergistic EZH2 inhibition and eccDNA targeting suppress AOM/DSS-induced CRC

**Fig. 8:**
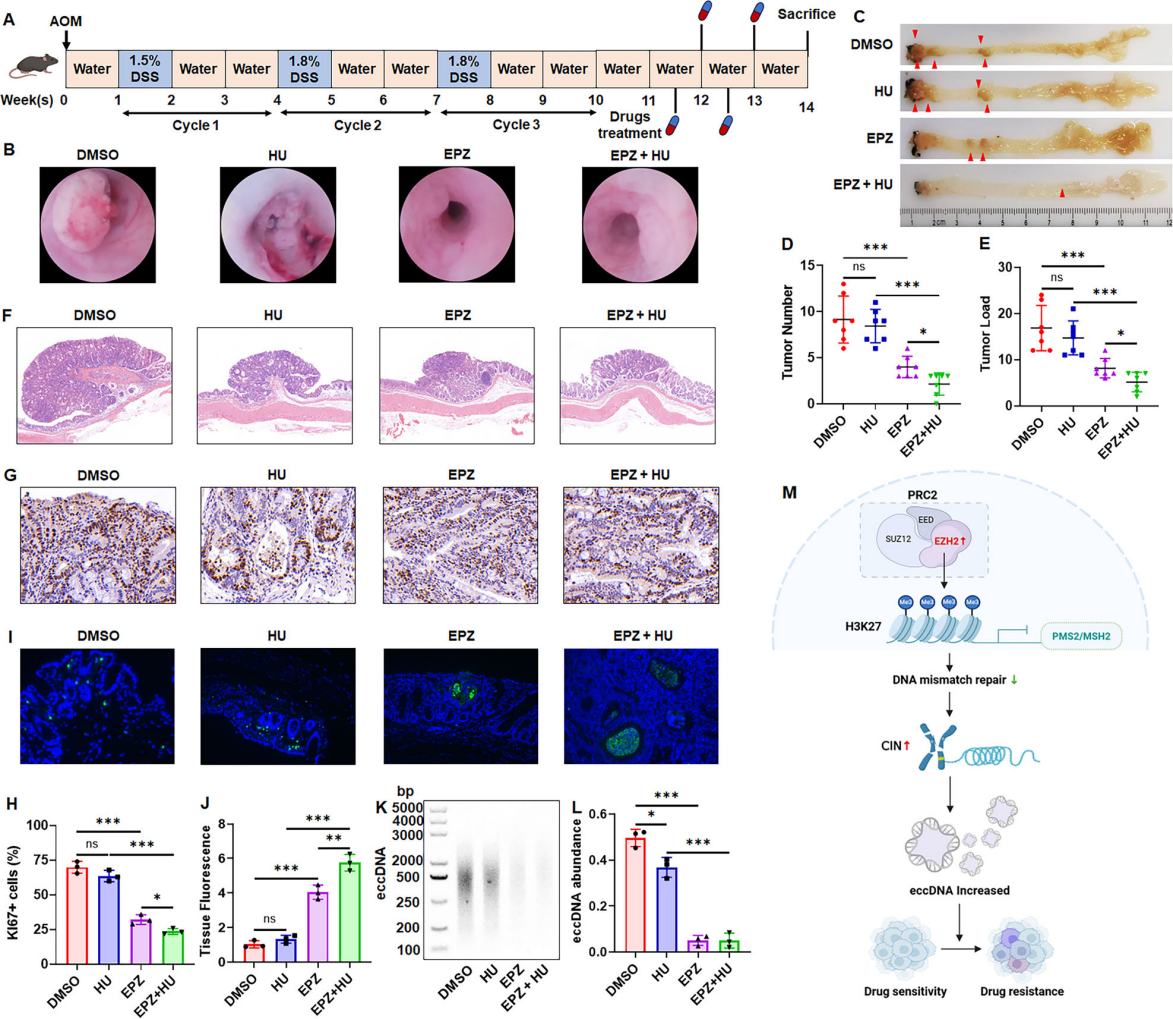
Combination of EZH2 inhibitor and hydroxyurea elicits anti-cancer effect in AOM/DSS-induced CRC. **A** Schematic of the experimental strategy for AOM/DSS-induced carcinogenesis and drug treatment. **B** Representative images showing colonoscopy evaluation of carcinogenesis after treatment with EPZ6438 (2 mg/kg, oral gavage twice per week) and HU (2 mg/kg, oral gavage twice per week). **C** Representative images of colorectal tumor burden in each group. **D** Colon tumor number in each group. **E** Colon tumor burden score in each group, three points for tumors larger than 3 mm in diameter, two points for diameters between 1 and 3 mm, and one point for <1 mm, the burden score was calculated as the sum of the above scores. **F** Representative images of H&E staining of the colons from AOM/DSS-induced carcinogenesis mice in each group. **G** Representative images of Ki-67 IHC staining in tumor sections. **H** Quantification of Ki-67 IHC staining in tumor sections. **I** Representative images of TUNEL fluorescence staining in tumor sections. **J** Quantification of TUNEL fluorescence staining in tumor sections. **K** EccDNA abundance after removal of linear DNA detected by agarose gel electrophoresis in each group. **L** Quantification of eccDNA agarose gel electrophoresis in each group. **M** Hypothesis Diagram, created with BioRender.com. Data were statistically analyzed using a Student *t* test. *p < 0.05, **p < 0.01, ***p < 0.001, ns: none significance.

To further dissect the involvement of eccDNA in tumor progression, a primary CRC model was developed by treating C57BL/6 mice with azoxymethane/dextran sodium sulfate (AOM/DSS). Treatment of the animals with EPZ6438, HU, or a combination of these agents was carried out for 14 days (Fig. 8A). HU, as a single-agent treatment, showed limited anti-tumor efficacy, while EPZ6438 resulted in visible anti-tumor activity (Fig. 8B, C). Remarkably, the combination treatment showed stronger synergistic effects (Fig. 8B, C), as evidenced by a substantial decrease in tumor incidence (Fig. 8D) and tumor burden (Fig. 8E). Immunohistochemical staining showed that EPZ6438 monotherapy significantly decreased Ki-67 expression levels, and that the combination therapy was able to suppress the proliferative marker even more completely (Fig. 8F–H). The TUNEL assays confirmed EPZ6438-mediated apoptosis, which was further potentiated by HU co-administration (Fig. 8I, J). Moreover, following combined treatment, agarose gel electrophoresis showed a significant decrease in eccDNA levels in the tumor tissues. These results indicate that the simultaneous blockade of EZH2 and eccDNA exerts a synergistic tumor-inhibitory effect.

## Discussion

The abnormal accumulation of eccDNA in MSI CRC is a sign of genomic instability that requires further examination. Mechanistically, a deficiency in MMR leads to a higher level of instability, which in turn supports the formation of eccDNA. When MMR fidelity is lost, the correction of DNA replication errors no longer takes place, thereby causing a persistent DNA damage response (DDR). This continuous DDR leads to the abnormal activation of alternative repair mechanisms, which results in widespread chromosomal breakage. These genomic fragments are subsequently circularized through nonhomologous end joining (NHEJ) or microhomology-mediated end joining (MMEJ), finally producing the eccDNA reservoir [16, 25, 26]. Additionally, fragile regions rich in microsatellites, which represent a hallmark of MSI tumors, are often the sites where DNA breaks occur [23, 25]. This observation prima facie further strengthens the rationale for their considerably greater load of eccDNAs relative to MSS tumors. On a clinical level, these eccDNAs are often found carrying major oncogenes and resistance determinants, thereby facilitating tumor progression and clinical heterogeneity. Our research reveals that eccDNA accumulation in MSI CRC is, at a molecular level, connected to the instability caused by MMR deficiency, where extendable insertion/deletion mutations are key contributors to eccDNA formation.

The CRC MSI subtype has different genomic instability features that relate to various molecular levels. At the chromosomal level, beyond characteristic microsatellite sequence length variations, we observed frequent numerical chromosomal abnormalities and complex structural rearrangements [2, 27]. These CIN events are mechanistically intertwined with eccDNA biogenesis. Specifically, chromosomal breakage provides the essential DNA substrates for eccDNA, while aberrant DNA repair pathways mediate their subsequent circularization. Furthermore, eccDNAs can interfere with mitotic processes directly and thus increase CIN, ultimately establishing a self-reinforcing feed-forward loop that leads to rapid tumor evolution [3, 24].

Besides innate epigenetic mechanisms, external factors, particularly the gut microbiota, may also enhance the EZH2–MMR–eccDNA axis. Accumulating evidence suggests that microbial metabolites and host–microbiota crosstalk can dynamically modulate histone landscapes and DNA repair fidelity in intestinal epithelial cells [28, 29]. Although no direct experimental data demonstrating microbiota-driven MMR silencing or eccDNA biogenesis have yet been reported, this is certainly a prime target for future studies. It is plausible that microbiota-derived signals cooperate with EZH2-mediated epigenetic regulation to determine the landscape of genomic instability in CRC. As the main catalytic element of PRC2, EZH2 is an essential factor in the initiation and development of MSI-H colorectal carcinomas. We found that EZH2 directs the formation of closed chromatin configurations at the promoters of major *MMR* genes by catalyzing the addition of H3K27me3 marks. As a result of this epigenetic silencing, the levels of repair proteins (including PMS2 and MSH2) decrease, which reduces the capacity of the cells to repair DNA damage. Interestingly, the epigenetic control exerted by EZH2 over the MMR system not only determines genomic stability but also influences tumor mutational patterns, thereby affecting the cellular response to specific therapeutic options [30].

HU is a powerful inhibitor of ribonucleotide reductase and leads to replication stress by depleting intracellular dNTP pools, thus limiting the anabolic phase of eccDNA synthesis and amplification. Its primary mode of action might be complex and multifactorial. Apart from just restricting nucleotide resources, HU causes significant replication fork stalling, which could even counterintuitively lead to a decrease in the rate of random DNA breakage events that act as critical initiators for eccDNA formation [31, 32]. Also, HU might affect the decision-making process of the cell in terms of choosing DNA repair pathways, which could result in a different balance between NHEJ and MMEJ (both pathways are important for eccDNA circularization) [31, 32]. Consequently, further studies are required to determine the precise molecular mechanisms underlying HU-mediated eccDNA suppression.

The mechanistic roles of eccDNA in tumor progression are quite diverse and intricate. From a genetic perspective, these extrachromosomal elements function as mobile units of genomic plasticity that undergo non-Mendelian, unequal distribution during mitosis, thereby fueling intratumoral heterogeneity [33]. The oncogenes encapsulated within these circles drive the constitutive activation of signaling pathways through high-copy-number focal amplification, while the amplification of drug resistance genes provides a rapid escape mechanism from therapeutic pressure [34, 35]. Furthermore, eccDNAs may act as molecular sinks, sequestering critical transcription factors through competitive binding and disrupting the stoichiometric equilibrium of established gene regulatory networks. Although our Circle-seq analysis identified a marked enrichment of oncogenic sequences within the eccDNA pool, precise stoichiometric quantification of their absolute copy number was not performed. Future investigations utilizing DNA fluorescence in situ hybridization (FISH) or digital PCR (dPCR) will be essential to ascertain the focal amplification architecture of these eccDNA-encoded oncogenes and to definitively assess their functional contributions to CRC progression.

The co-inhibition of EZH2 and eccDNA exhibits potent synergistic lethality through complementary molecular mechanisms. EZH2 inhibitors reactivate epigenetically silenced *MMR* genes, thereby partially restoring genomic integrity and suppressing de novo eccDNA biogenesis. Concurrently, eccDNA-clearing agents directly eliminate existing extrachromosomal circular DNA molecules, effectively extinguishing the constitutive oncogenic flux driven by eccDNA-encoded factors. This dual-pronged strategy simultaneously disrupts the epigenetic landscape and genetic volatility, creating a therapeutic bottleneck that hinders tumor adaptive evolution.

Our findings carry profound translational implications for the management of CRC. The quantitative burden of eccDNA in tumor tissues or systemic circulation may serve as a predictive biomarker to identify MSI-H CRC patients who are more likely to derive clinical benefit from EZH2-targeted therapies. The simultaneous targeting of EZH2 and the downstream products of genomic volatility (eccDNA) at two different points constitutes a sophisticated strategy to overcome therapeutic resistance in MSI-H CRC, a cancer type characterized by extraordinary genomic instability. The synergistic anti-tumor effects observed with EPZ6438 and HU in our preclinical models provide a strong rationale for the clinical development of such combinatorial regimens to improve patient outcomes.

In summary, this study demonstrates the potent therapeutic synergy of dual targeting of EZH2 and eccDNA in CRC. Along with restoring the balanced epigenetic state and removing the oncogenic extrachromosomal reservoir, this approach tackles the key factors of MSI-H tumor development. Besides introducing a novel concept of combination therapy for CRC, our results also outline a rationale for the application of eccDNA-targeted drugs in different types of malignancies.

## Supplementary information

original data

SUPPLEMENTAL MATERIAL

## Data Availability

The data presented in this study are available upon request from the corresponding author.
