# Supplementary material for: EZH2 deficiency suppresses colorectal cancer progression by inhibiting the mismatch repair pathway and consequently reducing extrachromosomal circular DNA formation
Source: Cell Death Dis. 2026 Jun 3;17(1):691. doi: 10.1038/s41419-026-08919-3 (PMC13443750; doi:10.1038/s41419-026-08919-3)
Supplement: Supplementary file 1 [file 41419_2026_8919_MOESM1_ESM.pdf]

Figure 4C

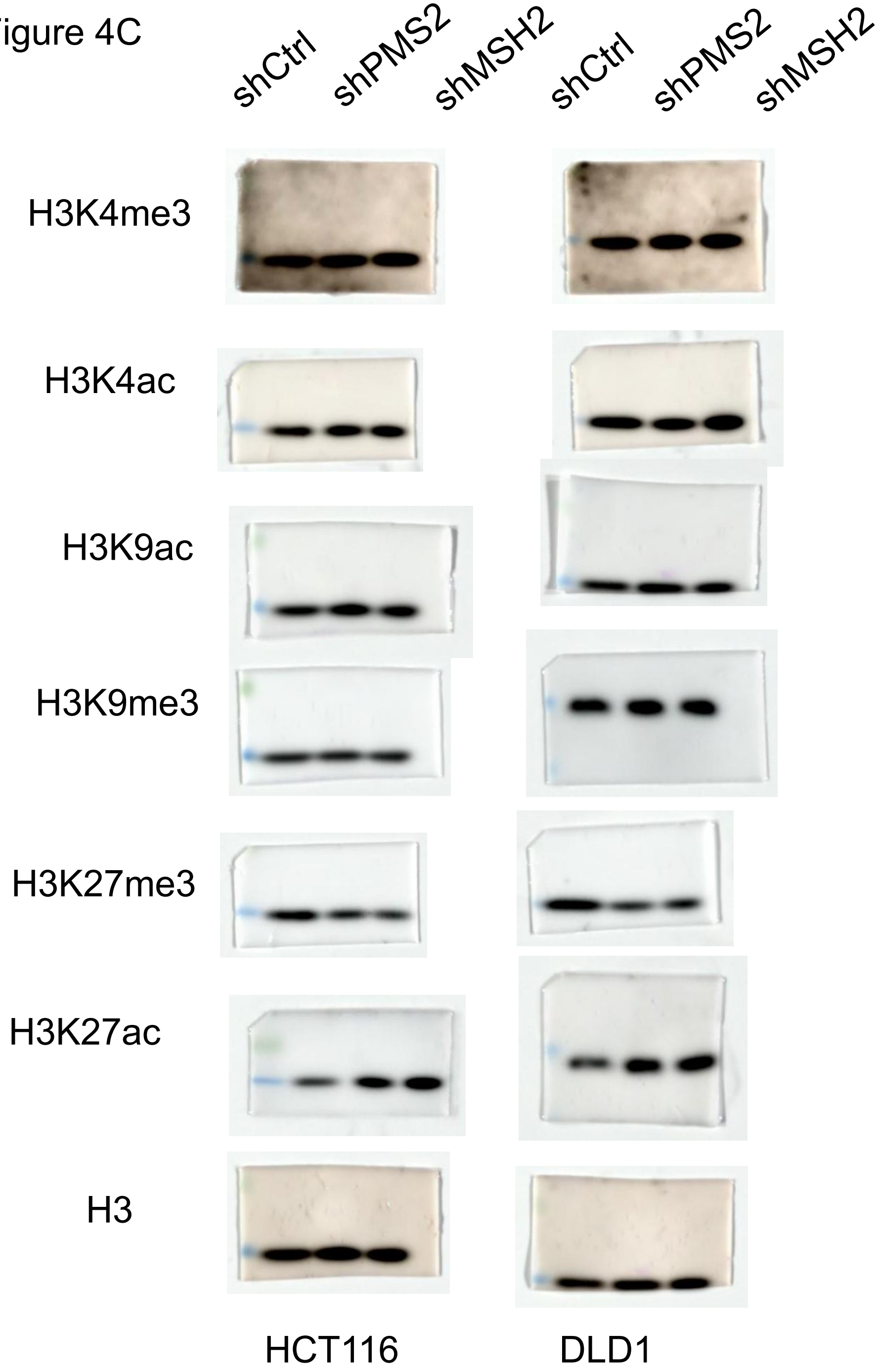

Figure 4F

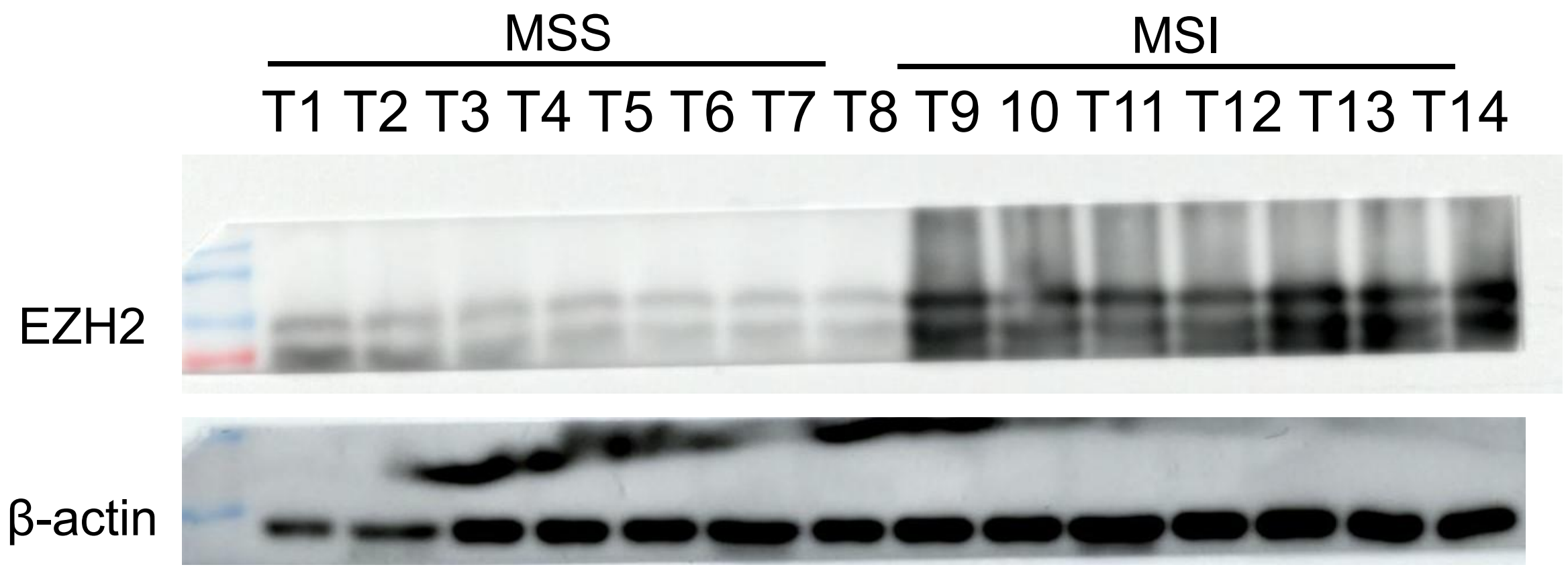

Figure 4G

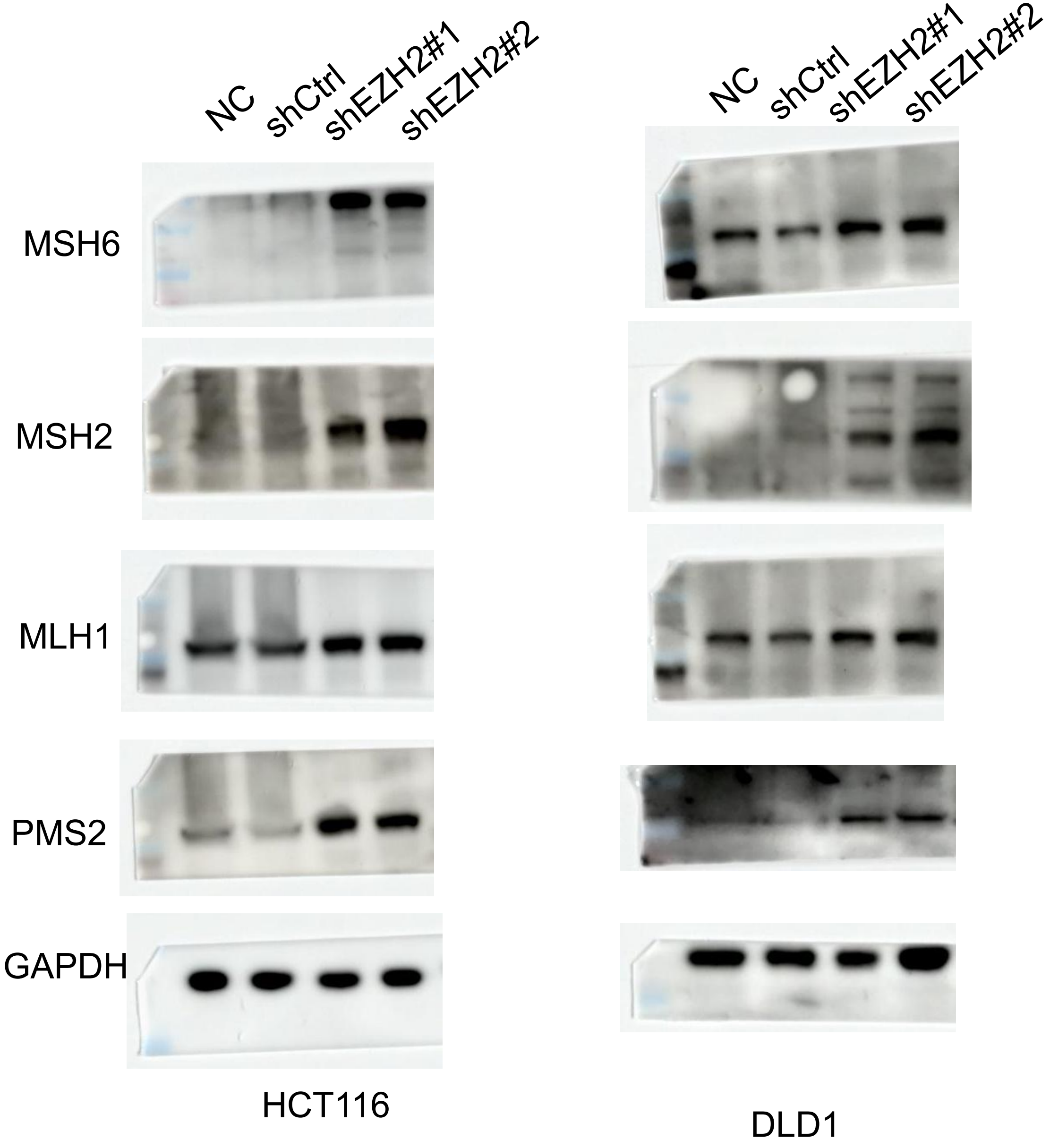
NC
shCtrl
shEZH2#1
shEZH2#2
MSH6
MSH2
MLH1
PMS2
GAPDH
HCT116
DLD1

Figure 6B

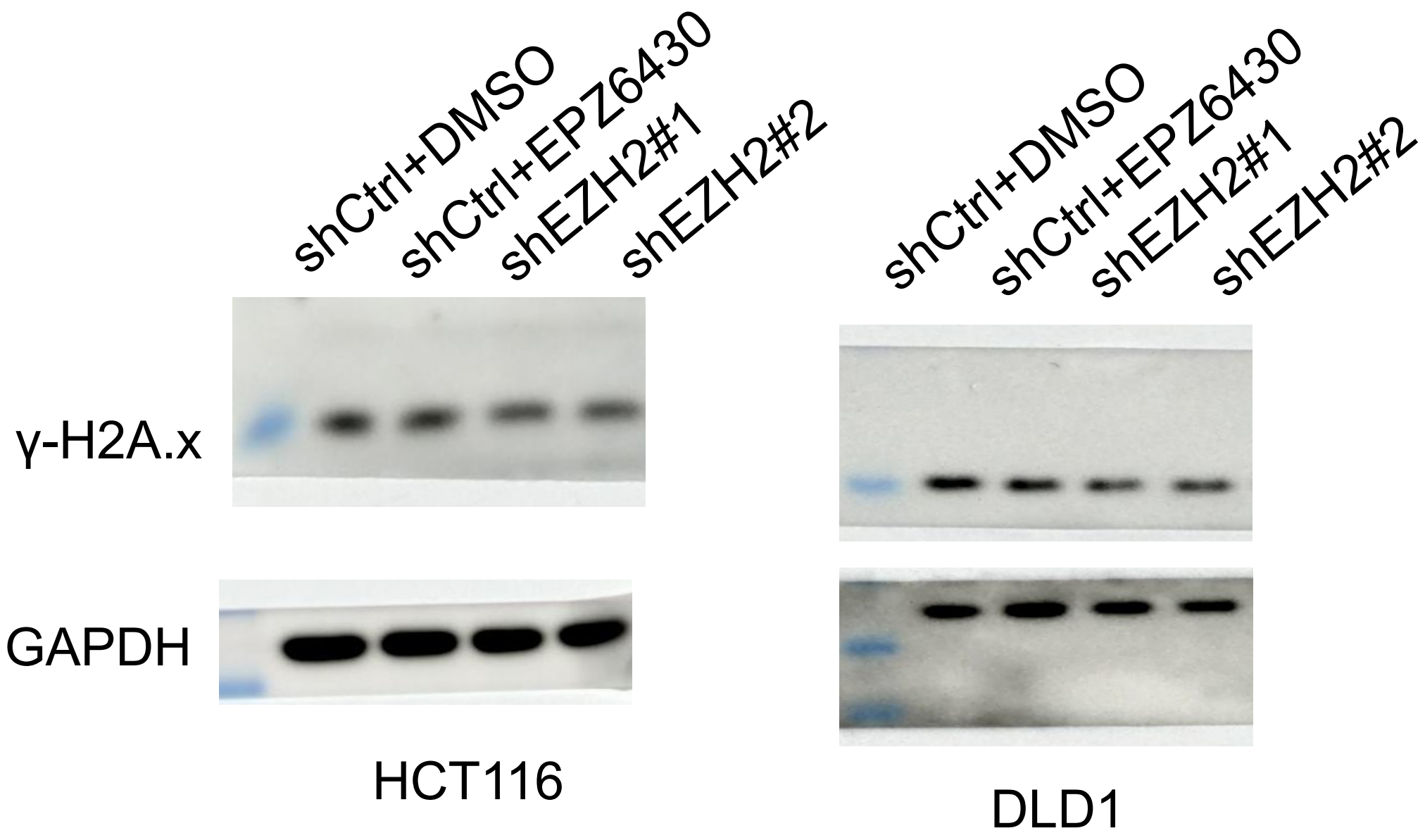

shCtrl+DMSO
shCtrl+EPZ6430
shEZH2#1
shEZH2#2
γ-H2A.x
GAPDH
HCT116
shCtrl+DMSO
shCtrl+EPZ6430
shEZH2#1
shEZH2#2
DLD1

S Figure 2A-C

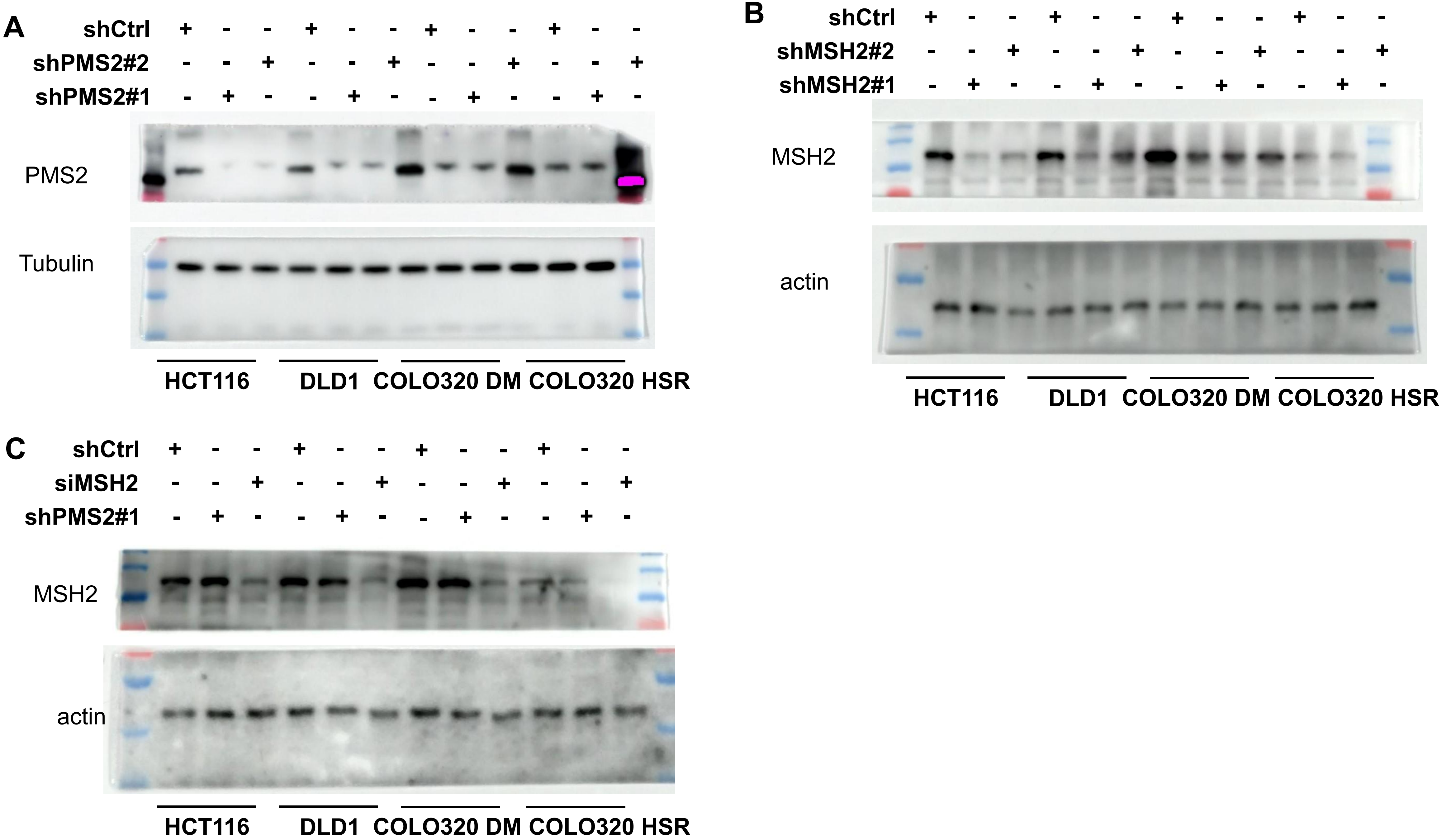
A
shCtrl + - - + - - + - - + - -
shPMS2#2 - - + - - + - - + - - +
shPMS2#1 - + - - + - - + - - + -
PMS2
Tubulin
HCT116
DLD1
COLO320 DM
COLO320 HSR
B
shCtrl + - - + - - + - - + - -
shMSH2#2 - - + - - + - - + - - +
shMSH2#1 - + - - + - - + - - + -
MSH2
actin
HCT116
DLD1
COLO320 DM
COLO320 HSR
C
shCtrl + - - + - - + - - + - -
siMSH2 - - + - - + - - + - - +
shPMS2#1 - + - - + - - + - - + -
MSH2
actin
HCT116
DLD1
COLO320 DM
COLO320 HSR

S Figure 4A

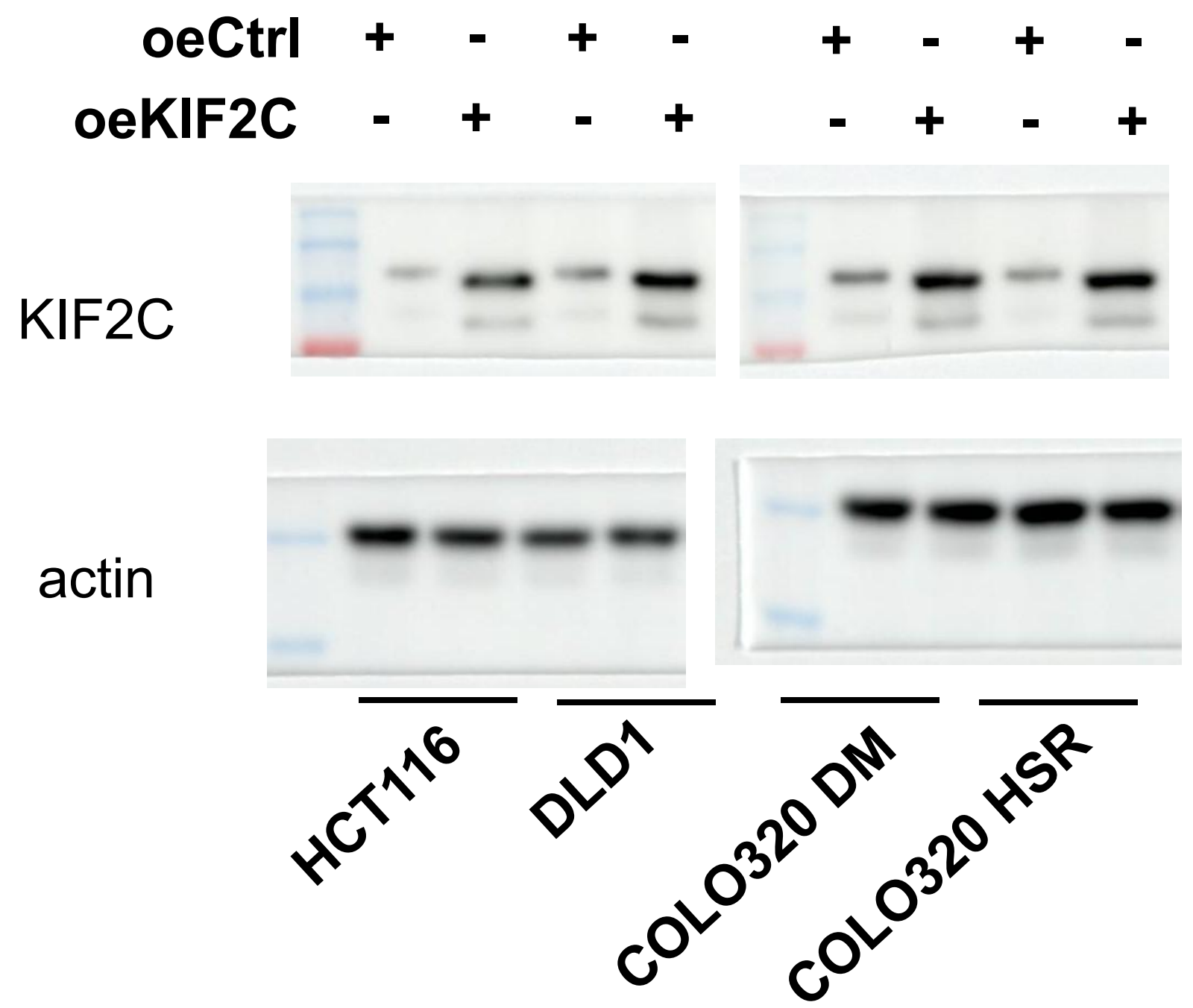

oeCtrl + - + - + - + -
oeKIF2C - + - + - + - +
KIF2C
actin
HCT116
DLD1
COLO320 DM
COLO320 HSR

PMS2

Primer1

重复1		shCtrl				shEZH2	
INPUT	18.53	18.22	18.21		18.16	18.21	18.05
IgG	26.01	25.62	25.45		25.23	25.13	25.16
H3K27me3	22.24	21.64	21.45		21.45	22.19	22.2
H3K27AC	22.4	22.17	21.74		21.88	21.67	21.47
重复2		shCtrl				shEZH2	
INPUT	17.25	17.42	17.3		17.42	17.55	17.23
IgG	25.23	25.08	24.8		25.66	25.31	25.37
H3K27me3	20.99	20.76	20.34		20.61	20.33	20.51
H3K27AC	21.59	21.5	21.22		21.94	21.62	21.54

Primer2

重复1		shCtrl				shEZH2	
INPUT	25.11	25.09	24.85		25.12	25.2	25.19
IgG	30.64	31.2	31.28		34.22	33.71	33.54
H3K27me3	28.77	28.41	28.13		28.53	29.03	28.87
H3K27AC	29.03	28.49	29.24		28.91	29.28	29.54
重复2		shCtrl				shEZH2	
INPUT	25.07	24.91	24.81		25.02	24.92	25.08
IgG	31.3	31.58	31.28		33.38	33.34	33.53
H3K27me3	28.65	28.44	28.65		28.7	28.68	28.52
H3K27AC	28.76	28.9	29.22		29.7	29.65	29.68

Primer3

重复1		shCtrl				shEZH2	
INPUT	17.14	17.12	17.1		17	17.04	16.95
IgG	36.18	36.26	34.55		33.07	33.73	33.15
H3K27me3	19.96	20.06	20.09		20.95	20.28	20.43
H3K27AC	21.07	21.35	21.21		21.15	21.07	20.94
重复2		shCtrl				shEZH2	
INPUT	17.22	17.28	17.19		17.06	17.1	17.18
IgG	38.01	35.34	34.21		32.96	31.57	30.81
H3K27me3	20.18	20.06	20.25		20.43	20.4	20.49
H3K27AC	21.18	21.45	21.33		21.24	21.32	21.2

Primer4

重复1		shCtrl				shEZH2	
INPUT	24.3	24.13	24.05		24.33	24.27	24.16
IgG	34.99	NaN	NaN		NaN	37.78	NaN
H3K27me3	26.74	26.6	26.5		27.37	27.52	27.64
H3K27AC	29.3	28.59	28.07		30.2	29.07	28.71
重复2		shCtrl				shEZH2	
INPUT	24.29	24.18	24.02		24.36	24.26	24.18
IgG	NaN	35.05	NaN		35.18	NaN	NaN
H3K27me3	26.75	26.56	26.67		27.52	27.78	27.55
H3K27AC	28.95	28.9	28.33		29.62	28.87	28.64

Primer5

重复1		shCtrl				shEZH2	
INPUT	24.27	24.18	24.2		24.45	24.59	24.91
IgG	31.24	NaN	NaN		31.45	38.01	34.66
H3K27me3	25.69	25.63	25.61		26.67	26.92	27.46
H3K27AC	28.98	28.66	29.37		29.14	30.04	29.58
重复2		shCtrl				shEZH2	
INPUT	23.97	23.8	23.81		24.02	24.71	25.12
IgG	34.12	NaN	30.76		NaN	NaN	35.69

H3K27me3	26	25.64	25.59		26.55	27.05	27.29
H3K27AC	29.13	29.2	28.56		29.42	29.83	30.38

MSH2

Primer1

重复1		shCtrl			shEZH2	
INPUT	25.57	25.56	25.83	25.52	25.73	25.47
IgG	34.2	33.32	33.31	32.76	NaN	32.32
H3K27me3	29	28.58	28.54	28.79	29.44	29.42
H3K27AC	28.54	29.75	29.69	30.4	29.18	30.88
重复2		shCtrl			shEZH2	
INPUT	24.89	25.15	25.02	25.32	25.22	25.16
IgG	34.02	34.58	33.89	31	31.92	32.08
H3K27me3	28.75	28.79	28.21	28.38	28.21	27.69
H3K27AC	29.05	29.09	28.51	29.97	29.76	29.59

Primer2

重复1		shCtrl			shEZH2	
INPUT	24.12	23.74	23.94	24.02	24.08	24.05
IgG	31.13	31.46	29.3	27.15	30.33	29.98
H3K27me3	27.38	26.37	27.02	27.18	27.21	27.13
H3K27AC	27.18	27.98	28.15	28.63	26.73	29.02
重复2		shCtrl			shEZH2	
INPUT	23.87	23.75	23.55	23.32	23.77	23.87
IgG	29.64	28.93	29.32	31.18	30.88	30.36
H3K27me3	26.05	25.86	26.55	26.37	26.06	26.24
H3K27AC	26.75	26.56	27.25	27.83	28.35	28.02

Primer3

重复1		shCtrl			shEZH2	
INPUT	23.48	23.2	23.38	23.82	23.6	24.13
IgG	NaN	29.73	31.45	NaN	NaN	NaN
H3K27me3	27.02	26.15	26.42	27.12	26.55	27.66
H3K27AC	27.41	27.74	29.52	28.41	29.2	28.42
重复2		shCtrl			shEZH2	
INPUT	23.34	23.26	23.21	26.92	26.3	26.56
IgG	29.83	30.16	NaN	34.08	33.48	33.71
H3K27me3	27.99	25.47	25.99	30.36	30.65	29.77
H3K27AC	27.33	27.5	27.49	31.19	30.84	32.5

Primer4

重复1		shCtrl			shEZH2	
INPUT	23.93	23.54	23.6	23.89	23.61	23.75
IgG	29.07	28.78	29.06	28.91	28.88	28.35
H3K27me3	26.44	26.39	26.3	27.36	27.64	27.37
H3K27AC	27.02	26.96	26.5	27.92	27.66	27.18
重复2		shCtrl			shEZH2	
INPUT	23.64	23.4	23.31	23.31	23.4	23.62
IgG	28.64	28.41	28.91	28.22	28.33	28.55
H3K27me3	26.1	26.06	26.12	27.21	27.68	27.29
H3K27AC	26.65	26.56	26.33	27.28	27.05	25.75

Primer5

重复1		shCtrl			shEZH2	
INPUT	23.86	23.81	24.07	23.99	24.1	24.42
IgG	29.1	29.13	29.17	29.08	29.12	29.79
H3K27me3	26.73	26.95	26.71	28.27	29.04	27.55
H3K27AC	27.19	26.52	27.07	28.14	28.26	28.5
重复2		shCtrl			shEZH2	
INPUT	23.65	23.85	23.47	23.57	24.1	24.35
IgG	28.37	29.08	29.02	28.5	29.55	29.64

H3K27me3	26.62	26.05	25.95		27.82	28.37	28.38
H3K27AC	26.67	26.82	27.15		28.33	28.19	28.56
