# Supplementary material for: EZH2 deficiency suppresses colorectal cancer progression by inhibiting the mismatch repair pathway and consequently reducing extrachromosomal circular DNA formation
Source: Cell Death Dis. 2026 Jun 3;17(1):691. doi: 10.1038/s41419-026-08919-3 (PMC13443750; doi:10.1038/s41419-026-08919-3)
Supplement: Supplementary file 2 [file 41419_2026_8919_MOESM2_ESM.pdf]

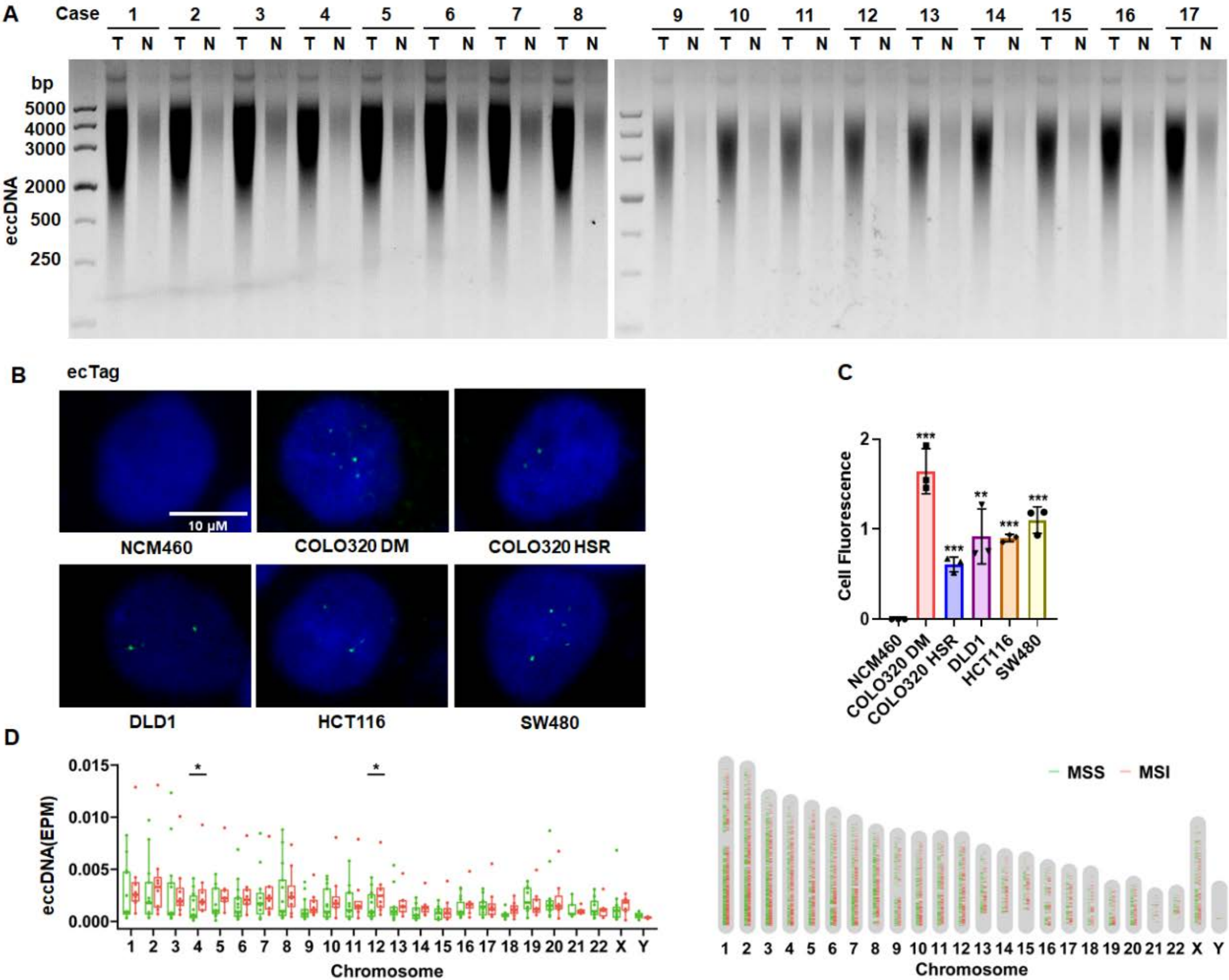
A
Case 1 2 3 4 5 6 7 8 9 10 11 12 13 14 15 16 17
T N
bp
5000
4000
3000
2000
500
250
eccDNA
B
ecTag
10 μM
NCM460
COLO320 DM
COLO320 HSR
DLD1
HCT116
SW480
C
Cell Fluorescence
0
1
2
***
**
NCM460
COLO320 DM
COLO320 HSR
DLD1
HCT116
SW480
D
eccDNA(EPM)
0.015
0.010
0.005
0.000
*
1 2 3 4 5 6 7 8 9 10 11 12 13 14 15 16 17 18 19 20 21 22 X Y
Chromosome
MSS
MSI

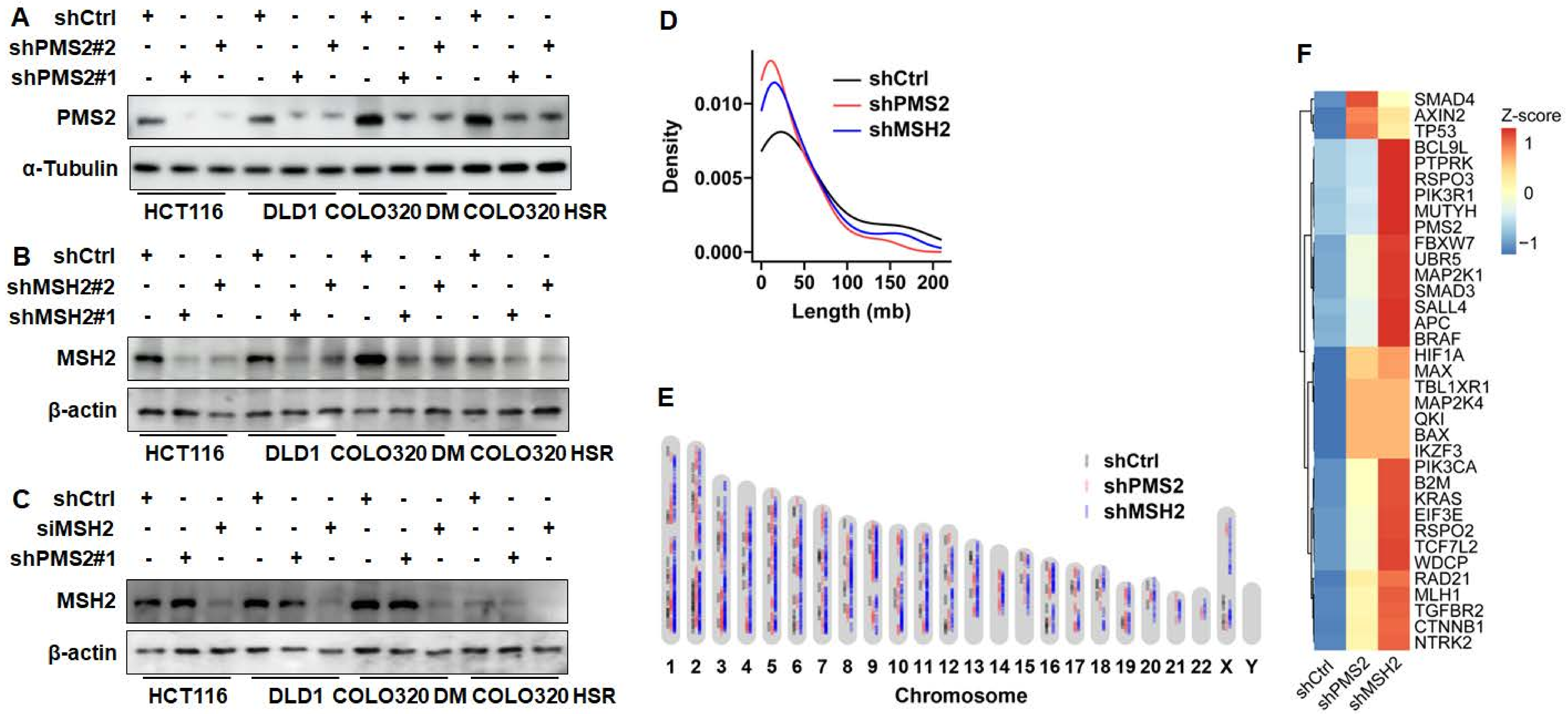

A
shCtrl
shPMS2#2
shPMS2#1
PMS2
α-Tubulin
HCT116
DLD1
COLO320 DM
COLO320 HSR
B
shCtrl
shMSH2#2
shMSH2#1
MSH2
β-actin
HCT116
DLD1
COLO320 DM
COLO320 HSR
C
shCtrl
siMSH2
shPMS2#1
MSH2
β-actin
HCT116
DLD1
COLO320 DM
COLO320 HSR
D
Density
Length (mb)
shCtrl
shPMS2
shMSH2
E
Chromosome
shCtrl
shPMS2
shMSH2
F
Z-score
SMAD4
AXIN2
TP53
BCL9L
PTPRK
RSPO3
PIK3R1
MUTYH
PMS2
FBXW7
UBR5
MAP2K1
SMAD3
SALL4
APC
BRAF
HIF1A
MAX
TBL1XR1
MAP2K4
QKI
BAX
IKZF3
PIK3CA
B2M
KRAS
EIF3E
RSPO2
TCF7L2
WDCP
RAD21
MLH1
TGFBR2
CTNNB1
NTRK2
shCtrl
shPMS2
shMSH2

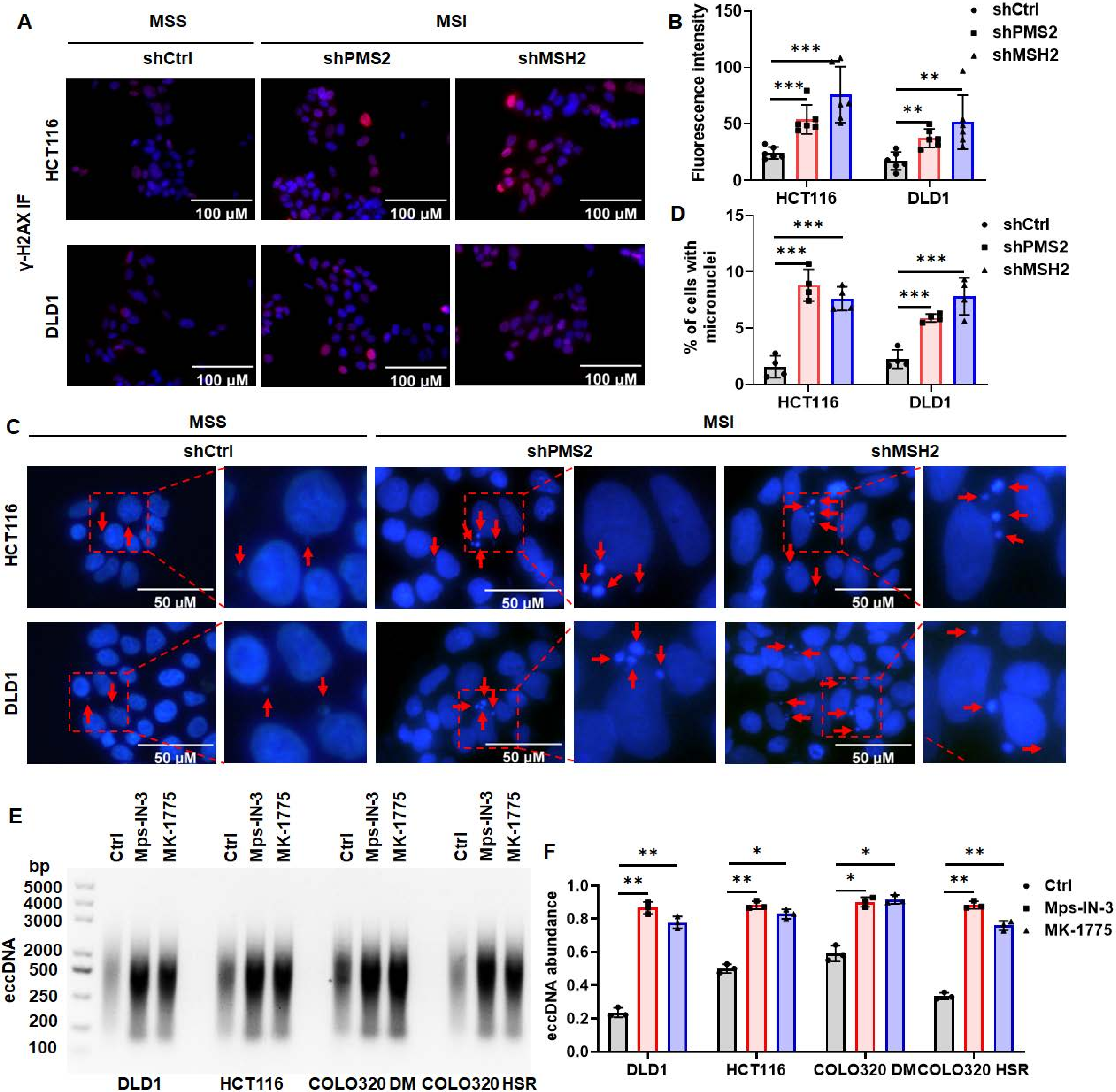
A
MSS
MSI
shCtrl
shPMS2
shMSH2
γ-H2AX IF
HCT116
DLD1
100 μM
B
Fluorescence intensity
shCtrl
shPMS2
shMSH2
HCT116
DLD1
D
% of cells with micronuclei
C
50 μM
E
bp
Ctrl
Mps-IN-3
MK-1775
eccDNA
DLD1
HCT116
COLO320 DM
COLO320 HSR
F
eccDNA abundance

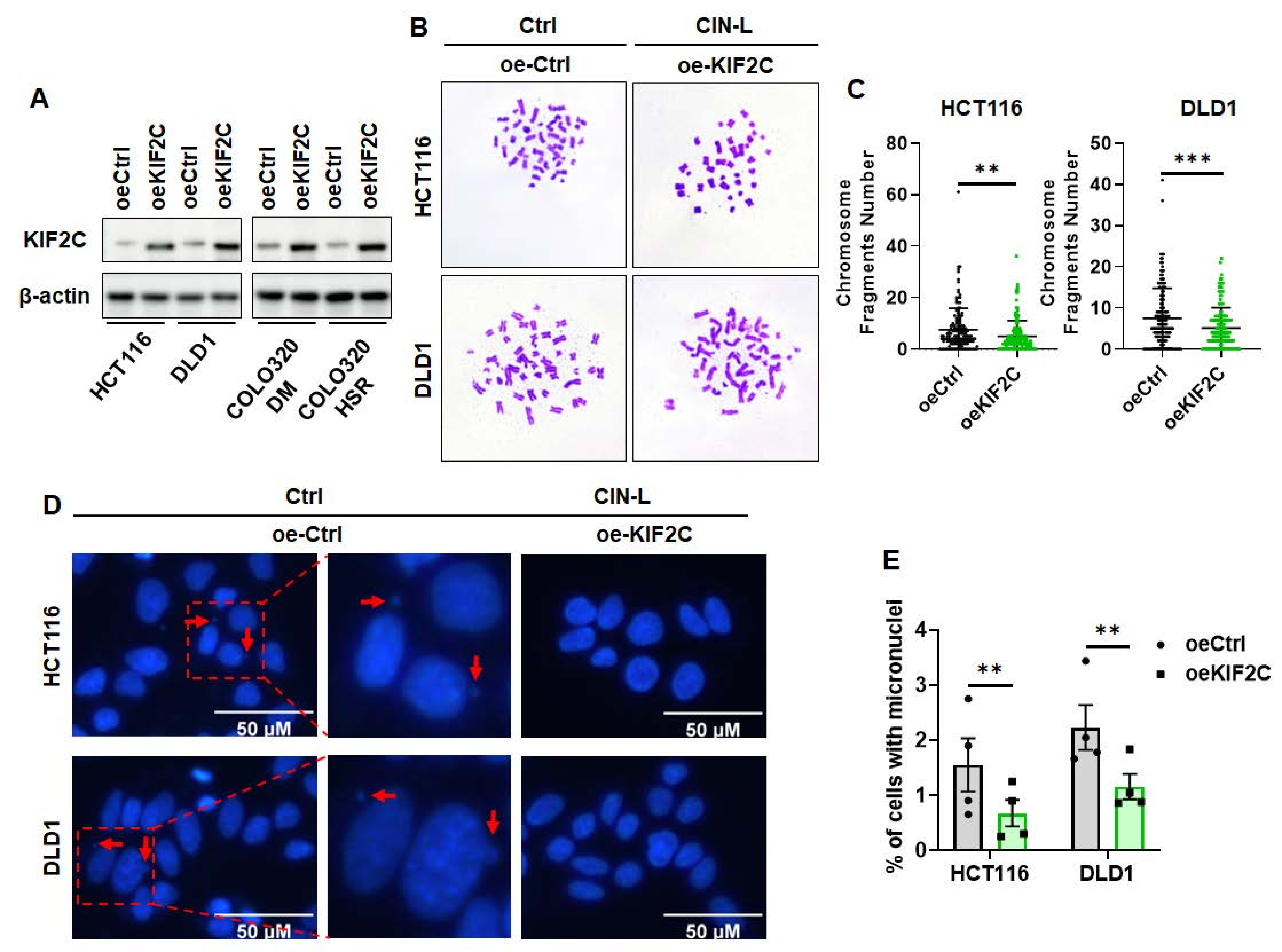

A
oeCtrl
oeKIF2C
oeCtrl
oeKIF2C
oeCtrl
oeKIF2C
oeCtrl
oeKIF2C
KIF2C
β-actin
HCT116
DLD1
COLO320 DM
COLO320 HSR
B
Ctrl
CIN-L
oe-Ctrl
oe-KIF2C
HCT116
DLD1
C
HCT116
DLD1
Chromosome Fragments Number
0
20
40
60
80
0
10
20
30
40
50
**
***
oeCtrl
oeKIF2C
oeCtrl
oeKIF2C
D
Ctrl
CIN-L
oe-Ctrl
oe-KIF2C
HCT116
DLD1
50 μM
50 μM
50 μM
50 μM
E
% of cells with micronuclei
0
1
2
3
4
**
**
HCT116
DLD1
oeCtrl
oeKIF2C

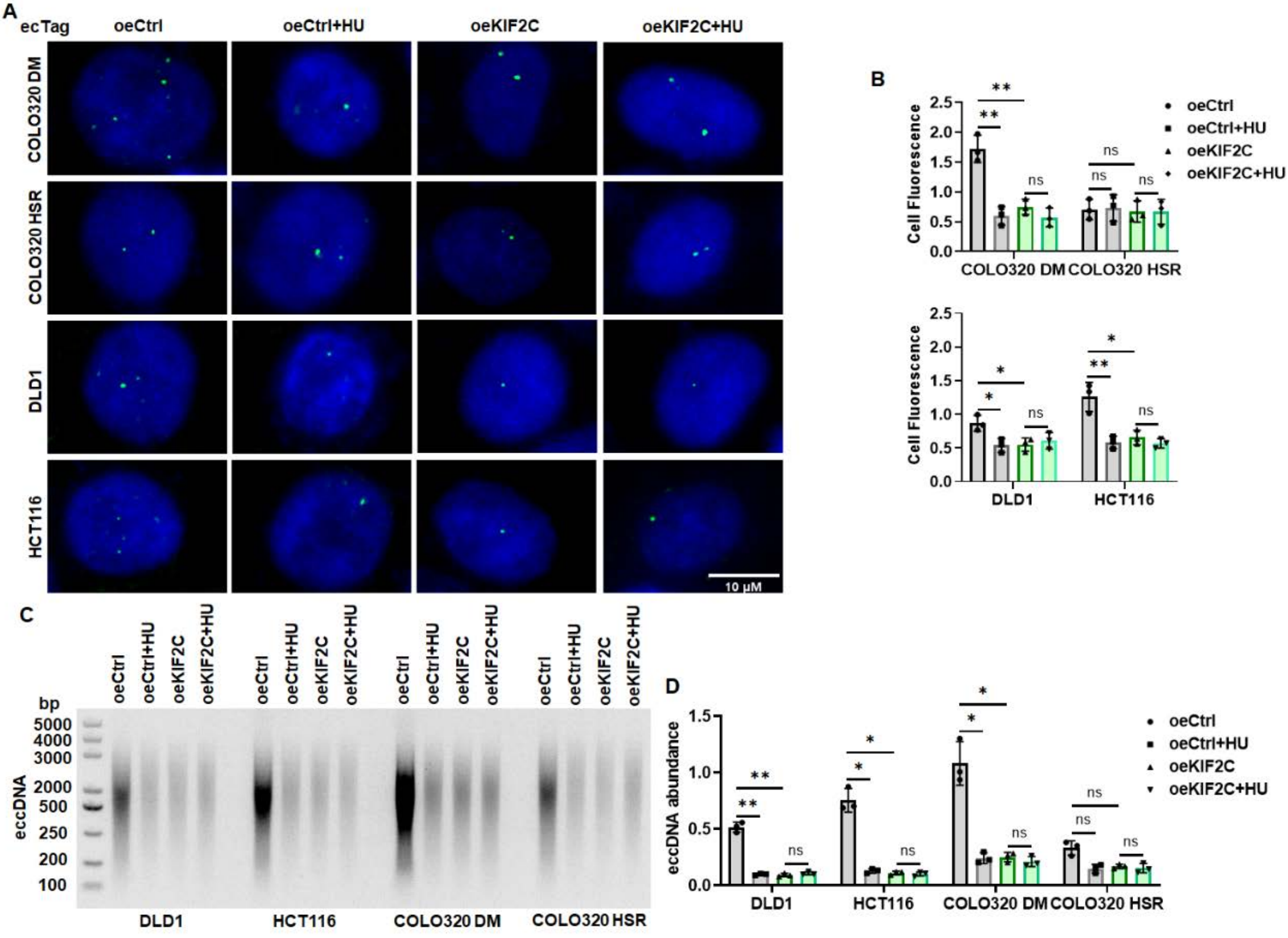
A
ecTag
oeCtrl
oeCtrl+HU
oeKIF2C
oeKIF2C+HU
COLO320 DM
COLO320 HSR
DLD1
HCT116
10 μM
B
Cell Fluorescence
oeCtrl
oeCtrl+HU
oeKIF2C
oeKIF2C+HU
COLO320 DM
COLO320 HSR
DLD1
HCT116
C
bp
eccDNA
5000
4000
3000
2000
500
250
200
100
DLD1
HCT116
COLO320 DM
COLO320 HSR
D
eccDNA abundance
oeCtrl
oeCtrl+HU
oeKIF2C
oeKIF2C+HU
DLD1
HCT116
COLO320 DM
COLO320 HSR

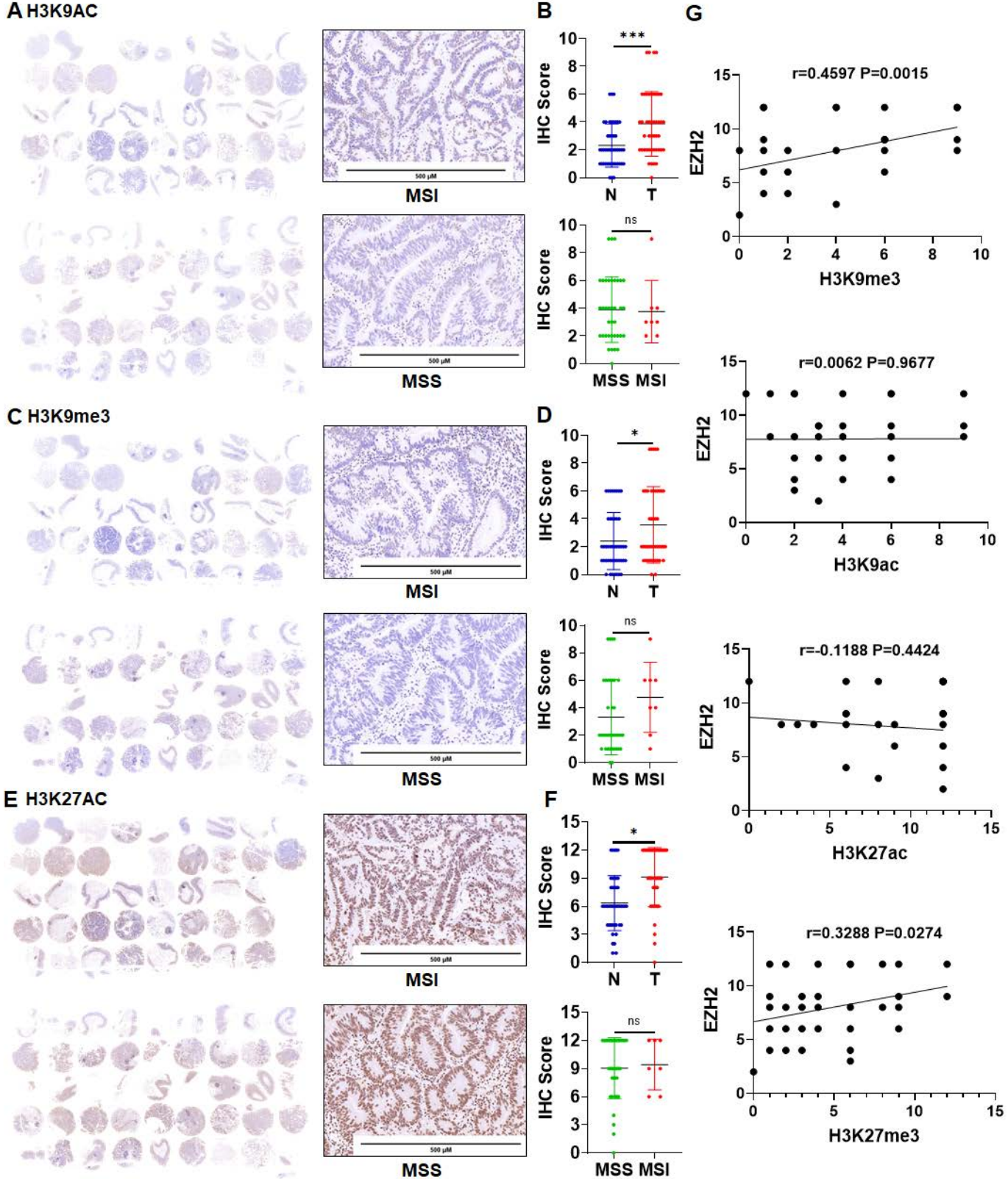
A H3K9AC
B
MSI
MSS
IHC Score
N
T
***
ns
MSS MSI
C H3K9me3
D
*
E H3K27AC
F
G
r=0.4597 P=0.0015
EZH2
H3K9me3
r=0.0062 P=0.9677
H3K9ac
r=-0.1188 P=0.4424
H3K27ac
r=0.3288 P=0.0274
H3K27me3
500 μM

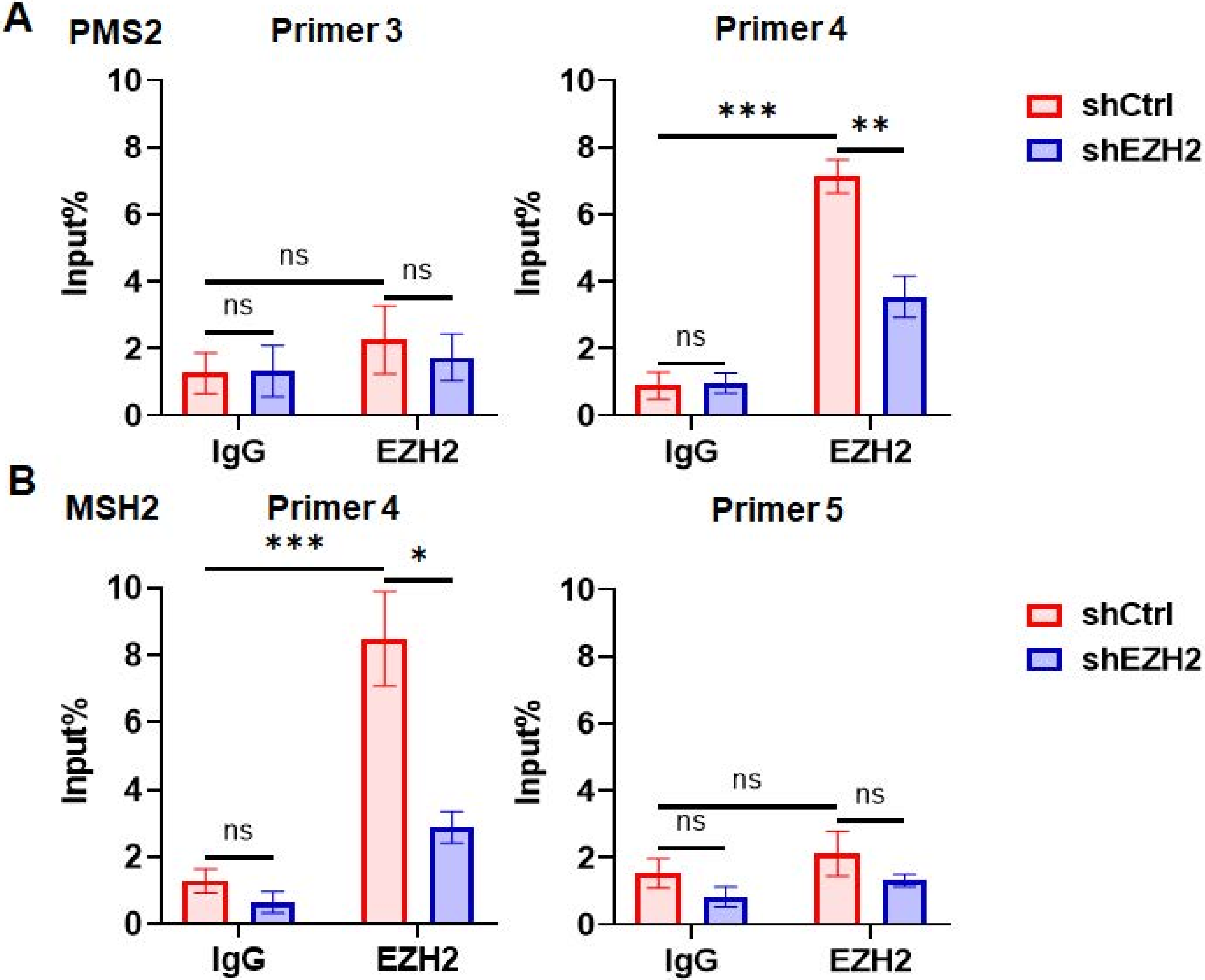
A
PMS2
Primer 3
Primer 4
Input%
10
8
6
4
2
0
ns
ns
ns
***
**
IgG
EZH2
shCtrl
shEZH2
B
MSH2
Primer 4
Primer 5
***
*
ns
ns
ns
ns

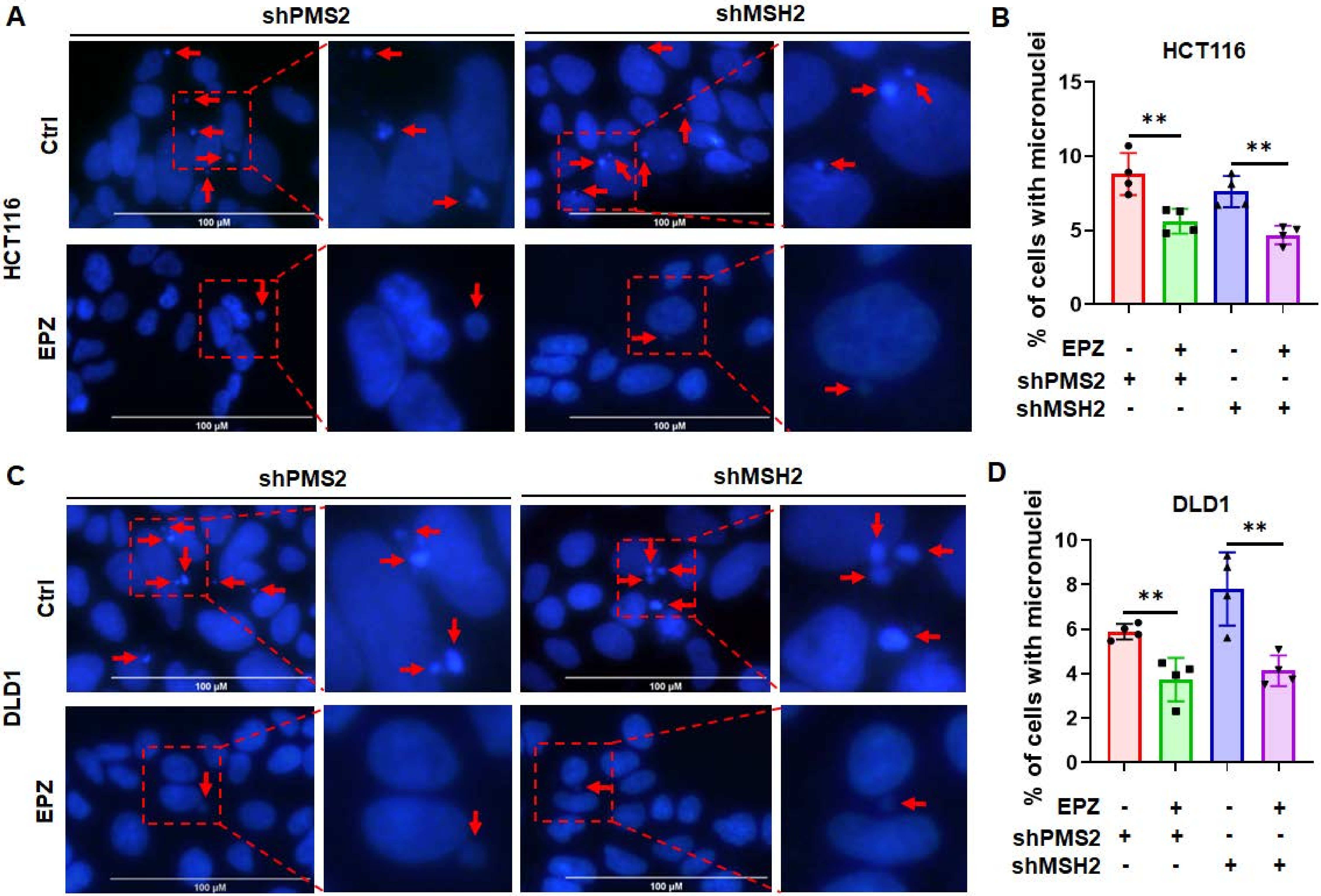

A
shPMS2
shMSH2
Ctrl
EPZ
HCT116
100 μM
B
HCT116
% of cells with micronuclei
15
10
5
0
**
**
EPZ - + - +
shPMS2 + + - -
shMSH2 - - + +
C
shPMS2
shMSH2
Ctrl
EPZ
DLD1
100 μM
D
DLD1
% of cells with micronuclei
10
8
6
4
2
0
**
**
EPZ - + - +
shPMS2 + + - -
shMSH2 - - + +

Supplementary Figure 1. EccDNA is ubiquitous in CRC. (A) EccDNA abundance after removal of linear DNA in CRC tumor and normal tissues detected by agarose gel electrophoresis. (B) Representative ecTag images in metaphase of NCM460, COLO320 DM, COLO320 HSR, DLD1, HCT116 and SW480 cell lines. The oligos target the junction of ecMAPK1. (C) Quantification of ecTag in metaphase of NCM460, COLO320 DM, COLO320 HSR, DLD1, HCT116 and SW480 cell lines. (D) Overall chromosomal distribution of eccDNA across the genome in MSI and MSS subtype CRC samples analyzed by Circle-seq. Data were statistically analyzed using a Student t test. $^{*}p < 0.05$, $^{**}p < 0.01$, $^{***}p < 0.001$, ns: none significance.

Supplementary Figure 2. Construction of MSI CRC models. (A) CRC cell lines with PMS2 knockdown by shRNA, Western Blot assay of PMS2 expression level. (B) CRC cell lines with MSH2 knockdown by shRNA, Western Blot assay of MSH2 expression level. (C) CRC cell lines with PMS2 knockdown by shRNA and MSH2 knockdown by siRNA, Western Blot assay of MSH2 expression level. (D) Density distributions of eccDNA in MSI-model cells (shPMS2 CRC cell lines and shMSH2 CRC cell lines) and wild type cells (shCtrl CRC cell lines) analyzed by Circle-seq. (E) Overall chromosomal distribution of eccDNA across the genome in MSI-model cells and wild type cells analyzed by Circle-seq. (F) Heatmap of differential expression of oncogenes in MSI-model cells and wild type cells analyzed by Circle-seq.Data were statistically analyzed using a Student t test. $^{*}p < 0.05$, $^{**}p < 0.01$, $^{***}p < 0.001$.

Supplementary Figure 3. Correlation between CRC subtypes and CIN. (A) Representative images of γ-H2AX IF staining in MSI-model cells and wild type cells. (B) Quantification of γ-H2AX IF staining in MSI-model cells and wild type cells. (C) Representative images of micronuclei DAPI staining in MSI-model cells and wild type cells. (D) Quantification of micronuclei DAPI staining in MSI-model cells and wild type cells. (E) EccDNA abundance after removal of linear DNA in metaphase of CRC cells after treatment with Mps-IN-3 and MK-1775 detected by agarose gel electrophoresis. (F) Quantification of eccDNA agarose gel electrophoresis in metaphase of CRC cells after treatment with Mps-IN-3 and MK-1775.Data were statistically analyzed using a Student t test. $^{*}p < 0.05$, $^{**}p < 0.01$, $^{***}p < 0.001$.

Supplementary Figure 4. Construction and Validation of CIN-L Model. (A) CRC cell lines with KIF2C overexpression, Western Blot assay of KIF2C expression level. (B) Representative images of karyotyping in CIN-L cells and wild type cells. (C) Numerical chromosome abnormalities in CIN-L cells and wild type cells. (D) Representative images of micronuclei DAPI staining in CIN-L cells and wild type cells. (E) Quantification of micronuclei DAPI staining in CIN-L cells and wild type cells. Data were statistically analyzed using a Student t test. $^{*}p < 0.05$, $^{**}p < 0.01$, $^{***}p < 0.001$.

Supplementary Figure 5. Enhanced chromosomal stability increases the abundance of eccDNA. (A) Representative ecTag images in metaphase of oeCtrl cells and oeKIF2C cells after treatment with HU. The oligos target the junction of ecMAPK1. (B) Quantification of ecTag in metaphase of oeCtrl cells and oeKIF2C cells after treatment with HU. (C) EccDNA abundance after removal of linear DNA in

metaphase of oeCtrl cells and oeKIF2C cells after treatment with HU detected by agarose gel electrophoresis. (D) Quantification of eccDNA agarose gel electrophoresis in metaphase of oeCtrl cells and oeKIF2C cells after treatment with HU. Data were statistically analyzed using a Student t test. *$p < 0.05$, **$p < 0.01$, ***$p < 0.001$, ns: none significance.

Supplementary Figure 6. Distinct histone modification patterns between MSI-H and MSS CRC. (A) Tissue chips analysis (left) and representative images (right) of H3K9AC IHC staining in CRC tumor and normal tissues. (B) H3K9AC IHC score in MSI and MSS CRC samples. (C) Tissue chips analysis (left) and representative images (right) of H3K9me3 IHC staining in CRC tumor and normal tissues. (D) H3K9me3 IHC score in MSI and MSS CRC samples. (E) Tissue chips analysis (left) and representative images (right) of H3K27AC IHC staining in CRC tumor and normal tissues. (F) H3K27AC IHC score in MSI and MSS CRC samples. (G) Correlation analysis of EZH2 and H3K9AC, H3K9me3, H3K27AC and H3K27me3 expression using tissue microarray. Data were statistically analyzed using a Student t test. *$p < 0.05$, **$p < 0.01$, ***$p < 0.001$.

Supplementary Figure 7. Validation of binding of EZH2 to the promoter region of PMS2 and MSH2 genes. (A) Validation of binding of EZH2 to the promoter region of PMS2 gene by CUT&RUN qPCR. (B) Validation of binding of EZH2 to the promoter region of MSH2 gene by CUT&RUN qPCR. Data were statistically analyzed using a Student t test. *$p < 0.05$, **$p < 0.01$, ***$p < 0.001$, ns: none significance.

Supplementary Figure 8. Validation of chromosomal stability by DAPI staining of micronuclei. (A) Representative images of micronuclei DAPI staining in HCT116 MSI-model cells and wild type cells. (B) Quantification of micronuclei DAPI staining in HCT116 MSI-model cells and wild type cells. (C) Representative images of micronuclei DAPI staining in DLD1 MSI-model cells and wild type cells. (D) Quantification of micronuclei DAPI staining in DLD1 MSI-model cells and wild type cells. Data were statistically analyzed using a Student t test. *$p < 0.05$, **$p < 0.01$, ***$p < 0.001$.

Supplementary Table 1. Basic clinical information from colorectal cancer patients.

Pathological diagnosis	Sample No.	Sample Type	Age	Height /cm	Weight /kg	Site	TNM stages	Clinical stages	MSI/ MSS
CRC	T1	Tumor	64	167	64	ascending	3/0/0	2	MSI
CRC	N1	Normal	64	167	64	ascending	3/0/0	2	MSI
CRC	T2	Tumor	50	170	75	ascending	3/2/0	3	MSI
CRC	N2	Normal	50	170	75	ascending	3/2/0	3	MSI
CRC	T3	Tumor	69	167	78	descending	3/1/0	3	MSI
CRC	N3	Normal	69	167	78	descending	3/1/0	3	MSI
CRC	T4	Tumor	85	162	58	ascending	4/2/0	3	MSI
CRC	T5	Tumor	53	155	55	rectum	4/0/0	2	MSI
CRC	T6	Tumor	51	160	55	ascending	3/1/0	3	MSI
CRC	T7	Tumor	62	155	55	rectum	3/1/0	3	MSI
CRC	T8	Tumor	40	160	55	rectum	4/1/1	4	MSI
CRC	T9	Tumor	55	165	66	ascending	3/1/0	3	MSS
CRC	N9	Normal	55	165	66	ascending	3/1/0	3	MSS
CRC	T10	Tumor	49	165	63	ascending	3/0/0	2	MSS
CRC	N10	Normal	49	165	63	ascending	3/0/0	2	MSS
CRC	T11	Tumor	45	162	56	ascending	3/0/1	4	MSS
CRC	T12	Tumor	67	158	52	sigmoid	3/1/0	3	MSS
CRC	T13	Tumor	83	167	61	ascending	3/2/1	4	MSS
CRC	T14	Tumor	70	175	60	sigmoid	3/2/1	4	MSS
CRC	T15	Tumor	55	165	79	sigmoid	3/0/0	2	MSS
CRC	T16	Tumor	58	178	78	descending	4/1/0	3	MSS
CRC	T17	Tumor	53	158	65	ascending	4/0/0	2	MSS
CRC	T18	Tumor	66	166	60	ascending	3/0/0	2	MSS
CRC	T19	Tumor	55	170	75	sigmoid	3/0/0	2	MSS
CRC	T20	Tumor	84	156	74	sigmoid	3/2/0	3	MSS
CRC	T21	Tumor	50	180	65	ascending	3/2/0	3	MSS
CRC	T22	Tumor	73	155	44	ascending	4/2/0	3	MSS
CRC	T23	Tumor	63	170	60	sigmoid	3/0/0	2	MSS

Supplementary Table 2. Summary of eccMAKP1 characteristics analyzed by Circle-map

GeneName	Sample	Chr	Start	End	Length	SplitReads	Score	Coverage
MAPK1	T2	22	20034441	31225185	11190744	2	54	2.2475
MAPK1	T3	22	20635465	29923825	9288360	2	52	2.3495
MAPK1	T6	22	11722826	39442854	27720028	41	938	9.09881
MAPK1	T7	22	19070376	25582029	6511653	4	200	4.58048
MAPK1	T7	22	20082248	38790344	18708096	3	144	3.6287
MAPK1	T7	22	21612901	44981805	23368904	6	189	3.62922
MAPK1	T8	22	19987956	31601570	11613614	3	97	2.57217
MAPK1	T11	22	17878101	38130772	20252671	8	292	3.03826
MAPK1	T17	22	17975206	49686005	31710799	2	128	3.70734
MAPK1	T17	22	20161047	44105222	23944175	7	322	3.67991
MAPK1	T17	22	20690131	24252781	3562650	3	58	4.36786
MAPK1	T18	22	18028080	36567952	18539872	2	69	9.48598
MAPK1	T18	22	18083341	33366320	15282979	2	76	10.0804
MAPK1	T23	22	21577270	25151033	3573763	4	100	1.27483
